# A novel and diverse group of *Candidatus* Patescibacteria from bathypelagic Lake Baikal revealed through long-read metagenomics

**DOI:** 10.1186/s40793-023-00473-1

**Published:** 2023-02-23

**Authors:** Jose M. Haro-Moreno, Pedro J. Cabello-Yeves, M. Pilar Garcillán-Barcia, Alexandra Zakharenko, Tamara I. Zemskaya, Francisco Rodriguez-Valera

**Affiliations:** 1 Evolutionary Genomics Group, Departamento Producción Vegetal y Microbiología, Universidad Miguel Hernández, Apartado 18, San Juan de Alicante, 03550 Alicante, Spain; 2 Cavanilles Institute of Biodiversity and Evolutionary Biology, University of Valencia, 46980 Paterna, Valencia Spain; 3 Instituto de Biomedicina y Biotecnología de Cantabria (IBBTEC), Universidad de Cantabria-Consejo Superior de Investigaciones Científicas, Santander, Spain; 4 Limnological Institute, Siberian Branch of the Russian Academy of Sciences, Irkutsk, Russia

**Keywords:** *Ca*. Patescibacteria, Lake Baikal, Long-read metagenomics, 16S rRNA

## Abstract

**Background:**

Lake Baikal, the world’s deepest freshwater lake, contains important numbers of *Candidatus* Patescibacteria (formerly CPR) in its deepest reaches. However, previously obtained CPR metagenome-assembled genomes recruited very poorly indicating the potential of other groups being present. Here, we have applied for the first time a long-read (PacBio CCS) metagenomic approach to analyze in depth the *Ca.* Patescibacteria living in the bathypelagic water column of Lake Baikal at 1600 m.

**Results:**

The retrieval of nearly complete 16S rRNA genes before assembly has allowed us to detect the presence of a novel and a likely endemic group of *Ca.* Patescibacteria inhabiting bathypelagic Lake Baikal. This novel group seems to possess extremely high intra-clade diversity, precluding complete genomes' assembly. However, read binning and scaffolding indicate that these microbes are similar to other *Ca.* Patescibacteria (i.e. parasites or symbionts), although they seem to carry more anabolic pathways, likely reflecting the extremely oligotrophic habitat they inhabit. The novel bins have not been found anywhere, but one of the groups appears in small amounts in an oligotrophic and deep alpine Lake Thun. We propose this novel group be named Baikalibacteria.

**Conclusion:**

The recovery of 16S rRNA genes via long-read metagenomics plus the use of long-read binning to uncover highly diverse “hidden” groups of prokaryotes are key strategies to move forward in ecogenomic microbiology. The novel group possesses enormous intraclade diversity akin to what happens with *Ca.* Patescibacteria at the interclade level, which is remarkable in an environment that has changed little in the last 25 million years.

**Supplementary Information:**

The online version contains supplementary material available at 10.1186/s40793-023-00473-1.

## Background

The Phylum *Ca.* Patescibacteria (formerly the Candidate Phyla Radiation, CPR) is a remarkably diverse set of bacteria discovered by single-cell amplified genomes (SAGs) [1] or metagenome-assembled genomes (MAGs) [2] with only a handful of cultivated representatives all of which seem to be parasites or epibionts of other bacteria or archaea [3–7]. They have been obtained from an extremely diverse set of habitats that range from aquatic [8–13], sediments (11) and soils [14, 15], to engineering environments [6, 7, 16, 17] and animal mucosas [18]. Many representatives within this phylum are anaerobic, at least partially, lacking key respiratory complexes while having the ability to perform mixed-acid fermentations [9, 19, 20]. However, some of us recently discovered the presence of abundant CPR genomes in a very stable and aerobic environment: the water column of the deep Lake Baikal in Siberia [11]. This gigantic Lake contains 20% of the liquid freshwater on Earth and its hypolimnion is always aerobic due to convection currents generated by the often colder surface [21]. The lake is frozen ca. 4–5 months per year, is extremely oligotrophic, and its microbiome has been recently studied by classical metagenomics adding 266 novel MAGs coming from it [11, 22]. In particular, 25 MAGs retrieved from 1250 and 1350 m deep samples belonged to CPRs, a remarkably high number for a freshwater oxic environment.

One problem with MAGs derived from Illumina reads is that they tend to be incomplete [23]. Specifically, rRNA genes assemble very poorly due to their conserved/variable nature [24]. Individual Illumina reads can be compared to 16S rRNA databases, which is routinely done to obtain community structure information about the sample, but with this approach novel microbes cannot usually be characterized due to the shortness of the individual reads. Recently, HiFi PacBio technology (among others) allows the generation of long metagenomic fragments that cover complete gene clusters that can be reliably annotated before assembly [23]. Furthermore, complete ribosomal operons can be retrieved allowing a very precise phylogenetic characterization of the microbes thus discovered.

Here we have sequenced by PacBio Sequel II HiFi technology a 1600 m sample from the Bathypelagic Lake Baikal that has revealed the existence of a novel, yet undescribed, clade of *Ca.* Patescibacteria. Analysis of their metagenomic bins indicates that this new clade is very diverse (probably at the level of new order or class). They possess the typical features of the *Ca.* Patescibacteria phylum such as lack of respiratory chains, lipid biosynthesis, etc., but consistently have a large number of amino acid biosynthetic routes not yet described in other members of the group. We propose for them the informal name Baikalibacteria.

## Materials and methods

### Sampling, DNA processing, and sequencing

Samples were taken at central Lake Baikal (53.17° N, 108.39° E) on 13 July 2019 (summer period) from the RV ‘Vereshchagin’ in July 2019 using a CTD-coupled rosette with sensors SBE 19 Plus and SBE 25 Sealogger CTD (Sea-Bird Electronics). Oxygen concentration was 12.2 mg/L (other metadata from the sample are available at [25]. A hundred (100) L sample was sequentially filtered through 20, and 0.22 μm pore size polycarbonate filters (Millipore). All filters were immediately frozen on dry ice and stored at − 80 °C until processing. The sediment sample was obtained by a core and the upper 5 cm was selected for DNA extraction. DNA extraction was performed from the 0.22 µm filter following the phenol: chloroform extraction [26]. The sediment metagenome, due to its low DNA concentration, was only sequenced using Illumina NextSeq (150 bp, paired-end reads). The bathypelagic 1600 m deep sample was sequenced using PacBio Sequel II (one 8 M SMRT Cell Run, 30-h movie).

### Raw read filtering and assembly of metagenomic samples

Illumina raw reads were trimmed with Trimmomatic v0.39 [27] and assembled using IDBA-UD v1.1 [28]. To improve the quality of the PacBio reads, we generated Highly Accurate Single-Molecule Consensus Reads (CCS reads) using the CCS v4.2 program of the SMRT-link package. We tweaked the program to get the CCS sequences that were produced with at least five raw PacBio reads (CCS 5). PacBio CCS reads were assembled using Flye v2.7 [29] with the metagenome option.

### Taxonomic and functional annotation of PacBio reads and assemblies

Prodigal v2.6.3 [30] was used to predict genes from the assembled contigs retrieved from the individual assembly, as well as from the PacBio CCS reads. tRNA and rRNA genes were predicted using tRNAscan-SE v2.0.5 [31] and barrnap v0.9 (https://github.com/tseemann/barrnap), respectively. Predicted protein-encoded genes were taxonomical and functionally annotated against the NCBI NR database using DIAMOND 0.9.15 [32] and against COG [33] and TIGRFAM [34] using HMMscan v3.3 [35].

16S rRNA gene sequences were retrieved from Illumina and PacBio reads. Candidate Illumina sequences in a subset of 20 million reads were extracted using USEARCH v6.1 [36] after an alignment against a non-redundant version of the SILVA database v138 [37]. Sequences that matched this database with an E-value < 10^–5^ were considered potential 16S rRNA gene fragments. Then, ssu-align v0.1.1 was used to identify true sequences aligning these candidate sequences against archaeal and bacterial 16S rRNA hidden Markov models (HMM). For the long-read sequences, candidate 16S rRNA sequences were extracted using barrnap from total PacBio CCS reads and determined as *bona fide* 16S rRNA gene sequences using ssu-align. The resulting 16S rRNA sequences (derived from short and long reads) were classified using the SINA algorithm [38] according to the SILVA taxonomy database (v 138.1). Illumina sequences were only classified if the sequence identity was ≥ 80% and the alignment length was ≥ 90 bp. Sequences failing these thresholds were discarded.

### Genome reconstruction

Assembled contigs longer or equal to 5 Kb were assigned to a phyla classification if at least 50% of the genes shared the same best-hit taxonomy. Contigs failing this threshold were grouped as unclassified. To bin the contigs into long-read metagenome-assembled genomes (LAGs), their taxonomic affiliation (including the unclassified) was used together with the principal component analysis of tetranucleotide frequencies, GC content, and coverage values within this sample and several metagenomic samples described in previous studies from the Lake Baikal [11, 22]. Tetranucleotide frequencies were computed using the wordfreq program in the EMBOSS package, and the principal component analysis was performed using the FactoMineR v1.42 package [39]. Coverage values were calculated by the alignment of metagenomic reads (in subsets of 20 million reads) against contigs using BLASTN v2.9.0 [40] (99% identity, > 50 bp alignment). Reads were normalized by the size of the contig in Kb and by the size of the metagenome in Gb (RPKGs). The degree of completeness and contamination of the resulting MAGs were estimated using CheckM v1.1.2 [41]. For the *Ca*. Patescibacteria LAGs, we used the 43 single-copy gene set proposed [2]. Average nucleotide identity (ANI) between MAGs and the reference genome was calculated using JSpecies v1.2.1 software with default parameters [42]. In the same way, average amino acid identity (AAI) among sequences was calculated using compareM (https://github.com/dparks1134/CompareM). Taxonomic classification of recovered MAGs was performed using the GTDB-Tk v 2.1.0 tool [43] using the Genome Taxonomy Database (GTDB) release R207 [44]. To check whether assembled genomes were complete (circular) we aligned the previous Illumina assemblies (MAGs) to find overlapping sequences at the ends of the PacBio assembly using BLASTN v 2.9.0, with a cutoff of 95%. The Illumina contig must align at least 250 bp to either sequence end to consider a match.

### Retrieval of *Ca.* Patescibacteria reference genomes

All *Ca.* Patescibacteria genomes available were downloaded from the NCBI database (January 2022). We included in the analysis a very recent set of *Ca.* Patescibacteria MAGs retrieved from diverse freshwater samples [12]. Sequences with contamination > 5% and/or completeness < 40% calculated by checkM v1.1.2 were discarded. Genomes were annotated following the same pipeline as “*Functional annotation of PacBio reads and assemblies*”. To remove redundancy, reference genomes were dereplicated using dRep v2.6.2 [45] using a threshold of 95% identity (species boundary).

### 16S rRNA phylogenetic tree of *Ca.* Patescibacteria

To characterize the diversity of *Ca.* Patescibacteria 16S rRNA sequences retrieved from the CCS reads (see above), we performed a maximum likelihood (ML) phylogenetic tree. Briefly, 16S rRNA sequences > 1000 nucleotides were selected and dereplicated by clustering them at 97% identity using cd-hit 4.8.1 [46]. We included in the phylogenetic analysis sequences retrieved from the CCS assembly as well as from the lake Baikal Illumina MAGs [11, 22], and the 16S rRNA gene sequences from the reference genomes described above. Sequences were aligned with muscle v3.8.1551 [47] and trimmed using trimAL v1.4.rev15 [48], ending with 1020 nucleotide positions in the alignment. The ML phylogenetic tree was constructed using IQ-TREE v1.6.12 [49] with the following parameters: ultrafast bootstrap (5000 replicates) [50] and the best fitted model GTR + F + I + G4.

### Metagenomic fragment recruitment

Metagenomes from different freshwater datasets were used to study the *Ca.* Patescibacteria distribution. Raw reads from Lake Baikal Illumina metagenomes [11, 22] were downloaded from the NCBI BioProject accession numbers PRJNA615165, PRJNA396997, and PRJNA521725. In addition, raw reads from a compendium of 119 metagenomic samples taken from 17 different freshwater lakes located in Europe and Asia were also retrieved from the European Nucleotide Archive (ENA) (PRJEB35640 and PRJEB35770) [12]. Before recruitment, the complete ribosomal rRNA gene operon was manually removed from each genome sequence. Metagenomic reads were trimmed using Trimmomatic v0.36 [27] and only those reads with a phred score ≥ 30, ≥ 50 bp long, and with no ambiguous bases (Ns) were kept. These high-quality metagenomic reads were then aligned using BLASTN [40], using a cut-off of 95% nucleotide identity over a minimum alignment length of 50 nucleotides. We required ≥ 70% of each genome to be covered by reads. They were used to compute the RPKG (reads recruited per Kb of genome per Gb of metagenome) values that provide a normalized number comparable across various metagenomes. Genomes that recruited less than three RPKG were considered not present in the sample. The average nucleotide identity based on metagenomic reads (ANIr) was calculated using BLASTN with a cutoff of 80% identity and a minimum alignment read of 50 bp.

### Read binning and recovery of Baikalibacteria sequences

In order to recover the unassembled diversity of *Ca*. Patescibacteria from the sample, CCS5 reads putatively classified to this phylum were grouped according to their coverage and composition using MetaBCC-LR [51], which allows the metagenomic binning of long-reads without a reference genome. We previously removed the sequences matching at high identity (97% identity) with the already assembled LAGs and Illumina MAGs. We consider a trinucleotide approach and the higher sensitivity (parameter value 10) to differentiate among highly divergent species. Sequences were subjected to a t-distributed stochastic neighbor embedding (t-SNE) to reduce dimensionality. The resulting bins were subjected to manual scrutiny to evaluate the accuracy of the method, analyzing the placement of the 16S rRNA genes on the phylogenetic tree. To obtain larger fragments by overlapping, CCS reads from individual read bins were subjected to an all-vs-all alignment using blastn (99% identity, > 1000 bp alignment end-to-end). Redundancy on each bin was removed using cd-hit [46], with the following options (nucleotide comparison, -G 0-aS 0.9-c 0.99-g 1).

### Metabolism inference from genomes

To determine the metabolic pathways present in the Baikalibacteria and other *Ca.* Patescibacteria genomes, proteins were aligned to the KEGG (Kyoto Encyclopedia of Genes and Genomes) using the kofam_scan (https://github.com/takaram/kofam_scan) tool [52]. The resulting KO numbers, considering only hits with an e value < 1e^−5^, were uploaded to the KEGG reconstruct pathway tool. Genes related to bacterioruberin biosynthesis were studied in more detail. Thus, protein sequences of the lycopene elongase (*lyeJ*), carotenoid 3,4-desaturase (*crt*D), and bisanhydrobacterioruberin hydratase (*cru*F) were downloaded from the NCBI protein repository and individually aligned using muscle [47]. Then, a custom HMM database was created to identify these genes on the genomes (e value < 1e^−15^) [53]. Maximum phylogenetic trees using iqtree (ultrabootstrap of 5000 replicates, autodetection of the best protein model) were performed for the retrieved LyeJ, CrtD, and CruF sequences.

## Results

### Bathypelagic Lake Baikal PacBio metagenome

Complementing previous Illumina bathypelagic datasets from Lake Baikal collected during summer and winter seasons at 1250 and 1350 m deep [11, 22], here we performed long-read metagenomic sequencing using PacBio CCS of a deep sample (1600 m) collected during the summer. A total of 90 Gb of raw data were sequenced (4.46 Gb after CCS 5 correction, see methods), with an average read size of 14.3 Kb (Additional file 1: Table S1). Given the proximity to the bottom (ca. 20 m deeper), we have also performed a standard (Illumina) metagenome from the upper 5 cm of a Baikal sediment sample. A principal coordinate analysis following a Bray–Curtis similarity clustering of the metagenomic reads showed that Lake Baikal sediment had little to no impact on the 1600 m water column sample, which was, on the other hand, quite similar to the 1250 and 1350 m samples collected during a previous summer period (Additional file 1: Fig. S1A) despite the change in sequencing technology. Using unassembled 16S rRNA gene PacBio reads, we observed that the 1600 m sample did not significantly change the global picture of the Baikalian bathypelagic microbiome at high-level taxonomic classification (i.e., phyla and classes) retrieved before by using the ca. 150 nucleotides Illumina reads (Additional file 1: Fig. S1B).

However, there were large changes in the proportions of some groups. Thus, it was noticeable a larger fraction of Actinobacteria (51.7%) (almost twice as much as detected at 1350 m) followed, in the second place, by *Ca.* Patescibacteria (15.2%), which were three times less abundant than estimated from the Illumina 1250–1350 m 16S rRNA gene fragments. It was also remarkable the smaller numbers of Crenarchaeota (1.1%) and Nitrospirota (0.21%), which were in a lower proportion (ca. 10–20 times) in the PacBio 1600 m metagenome (Additional file 1: Fig. S1B and C). We note that the observed microbial community differences can be caused by differences in (i) sample depth (1350 versus 1600 m) and (ii) 16S rRNA gene fragment length (ca. 100 bp from short-read and 1000 bp from long-read metagenome sequencing). However, a recent similar comparison carried out with surface samples of similar depth in Lake Baikal (unpublished data) and the Mediterranean Sea [23] did not reveal large differences in changing the sequencing technologies. Therefore, it is likely that the deeper sample has a larger proportion of *Ca.* Patescibacteria and Actinobacteria and fewer ammonia-oxidizing consortia.

### *Ca.* Patescibacteria 16S rRNA genes from Lake Baikal

**Fig. 1:**
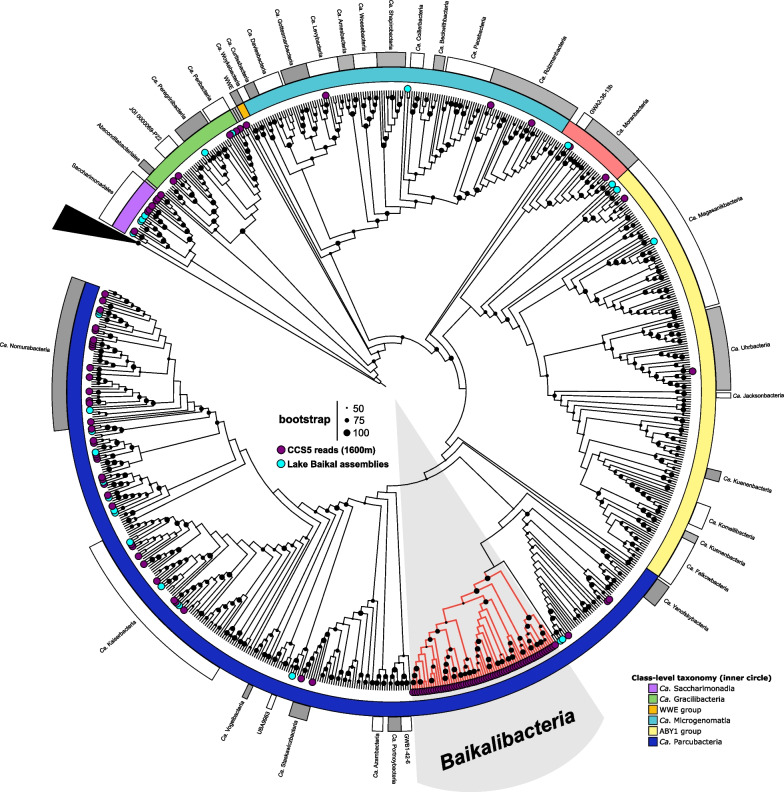
Phylogenetic tree (ML) of dereplicated (97%) 16S rRNA gene sequences from *Ca*. Patescibacteria retrieved from NCBI genome collection (January 2022) and freshwater datasets. MAGs coming from from Baikal 1250 m, 1350 m (Illumina) and 1600 m (PacBio CCS) are blue coloured. 16S RNA genes obtained from single reads from PacBio CCS from the 1600 m sample are purple coloured. Taxonomic classification based on the Genome Taxonomy Database (GTDB) is shown: inner circle—class, outer circle—family. The Baikalibacteria are highlighted in red

In order to classify in a finer detail the *Ca*. Patescibacteria of the bathypelagic Lake Baikal, we extracted and classified all the 16S rRNA sequences from the previous assemblies [11], together with those sequences from publicly available MAG and Long-read assembled genomes (LAGs, see below). We included in the analysis the 16S rRNA sequences from the CCS reads without any assembly (minimum size 1000 nucleotides). The resulting phylogenetic tree (Fig. 1) shows that *Ca*. Patescibacteria Lake Baikal MAGs and LAGs 16S rRNA gene sequences fell largely within already known groups. However, the phylogenetic tree also reveals a deep branch containing about half of the *Ca.* Patescibacterial 16S rRNA CCS reads (149 out of 278) without any close known clade (Fig. 1), indicating the presence of an abundant and novel family within the *Ca.* Paceibacteria class in the 1600 m sample. We will refer to this novel group by the informal name Baikalibacteria due to their abundance and apparent endemism in this lake (see below).

### Long-read-metagenomic assembled genomes (LAGs)

To further characterize the microbiome of Lake Baikal we assembled the 1600 m CCS 5 reads, resulting in 0.192 Gb of sequences (largest contig size: 2.26 Mb; average contig size 65.9 Kb) (Additional file 1: Table S1). Although the figure seems modest, the contigs produced recruited > 50% of the PacBio metagenome indicating that many of the individual reads assembled into contigs. Additionally, contig binning resulted in 56 LAGs > 50% complete (mean completeness 78.3%) and < 5% contaminated (Additional file 1: Table S2). Of the 56 LAGs, 32 had been already recovered as MAGs (> 99.5% average nucleotide identity [ANI]) by using the previously sequenced 1250 and 1350 m deep Illumina metagenomes [11]. However, LAGs were composed of larger contigs (8 times larger on average) and were slightly more complete (~ 12%) (Additional file 1: Table S3).

Among these, a total of 18 LAGs could be classified as members of the *Ca.* Patescibacteria phylum (Additional file 1: Table S2). They belonged to the classes *Ca.* Ardersenbacteria (n = 1), *Ca.* Gracilibacteria (n = 2), *Ca.* Microgenomatia (n = 1), *Ca.* Paceibacteria (n = 10), and *Ca.* Saccharimonadia (n = 3). One genome (*Ca.* Patescibacteria Baikal1600m-PB-G49) had no classification by the GTDB, which may indicate a novel class within the phylum *Ca.* Patescibacteria but we did not pursue this line here. A total of nine CPR LAGs were obtained in a single contig (Additional file 1: Table S2). We checked whether these genomes represented real circular closed genomes by using Illumina contigs that covered the ends of our PacBio assembly. Thus, we could prove that nine patescibacterial genomes were circular and complete (Additional file 1: Table S2, Fig. S2).

However, metagenomic recruitment at the species level (> 95%) showed that only 2.2% of the reads aligned to formerly assembled *Ca.* Patescibacteria MAGs [11] or the newly assembled LAGs. Recruitment values did not improve after lowering to the genus threshold (70–75% identity), with barely 2.4% of the reads matching *Ca.* Patescibacteria MAGs or LAGs, confirming the 16S rRNA picture that indicated most CPR in the sample belonged to the novel Baikalibacteria. The results also point to the well-known fact that some very abundant microbes assemble poorly due to high intraclade diversity, being marine Pelagibacterales and picocyanobacteria some of the most prominent examples (20).

### Baikalibacteria pangenome bins

To improve our understanding of this new group we binned together all PacBio reads classified as *Ca.* Patescibacteria (see methods) using the Baikalibacteria 16S rRNA containing reads as seeds. This allowed us to get individual bins each with consistent genomic parameters (GC content and tetranucleotide frequencies). Thus, 141.8 Mb of CCS reads not present in the LAGs were grouped into 14 read bins following this method (Additional file 1: Table S4). However, there was a very uneven distribution of the contigs containing Deep Baikal 16S rRNA genes, and four read bins (namely RBin04, RBin08, RBin09, and RBin10) contained nearly 70% of all their 16S rRNA sequences. Thus, we focused on these four bins to infer some of the ecological properties predicted from their pangenomes.

**Fig. 2:**
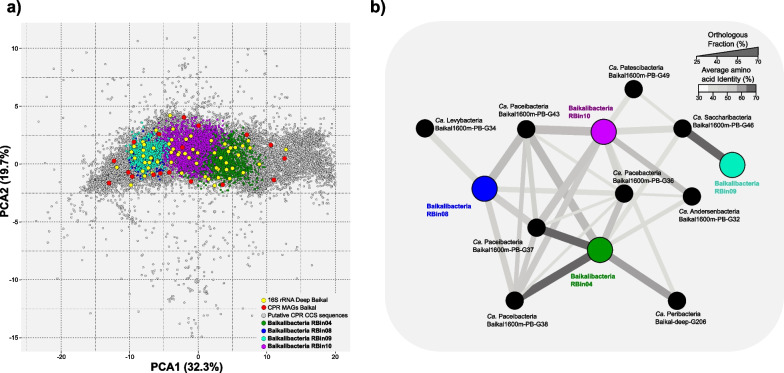
**a** Principal component analysis of tetranucleotide frequencies of sequences affiliated to *Ca.* Patescibacteria. Sequences affiliated to Baikalibacteria bins are colored in green, dark and light blue, and purple, respectively. As reference, we included in the comparison the MAGs retrieved from Lake Baikal Illumina (1250 and 1350 m) and PacBio (1600 m) metagenomes. CCS reads encoding for a Baikalibacteria 16S rRNA gene (Fig. 1) are colored in yellow. **b** Network graph showing the relationship between Lake Baikal MAGs and Baikalibacteria bins. The thickness and color of the line indicate the percentage of shared proteins (OF) and the mean amino acid identity (AAI), respectively. Interactions lower than 25% OF and/or 30% AAI were removed

A principal component analysis of all sequences showed the clear separation and clustering of the Baikalibacteria (Fig. 2A). These four groups were compared against Lake Baikal *Ca.* Patescibacteria LAGs to determine their relatedness and/or novelty at the genome level. In many cases, Baikalbacteria bins and LAGs share about 50–60% of their proteins, as indicated by the thickness of the line among genomes in the network in Fig. 2B. Thus, the novelty of the Baikalibacteria pangenomic bins was obvious for most of the comparisons, with average amino acid sequence identity (AAI) fluctuating between 40 and 50%. Only RBin09 and RBin04 showed a significant similarity (~ 65% AAI) to *Ca.* Saccharibacteria-Baikal1600m-PB-G48 and *Ca*. Paceibacteria-Baikal1600m-PB-G38, respectively. There was an agreement between the bin clusters and the phylogenetic placement of the ascribed 16S rRNA genes detected within them (Additional file 1: Fig. S3A). There was little to no synteny among CCS reads containing the 16S rRNA gene which is not surprising given that *Ca.* Patescibacteria in general contain their rRNA genes scattered throughout their genomes (Additional file 1: Fig. S3B).

Despite the considerable size (see Additional file 1: Table S4) of Baikalibacteria bins, we could not get any reconstructed genome (MAG) by assembly, and only a few, but larger, fragments could be recovered after read-to-read scaffolding. As mentioned before, this is not unusual for very abundant microbes containing high intrapopulation diversity, such as *Pelagibacter* and *Prochlorococcus* [23, 54]. To assess whether this was the case for Baikalibacteria, we compared the average nucleotide identity based on metagenomic reads (ANIr) of the four read bins with those of other LAGs. Indeed, the values for Baikalibacteria were much lower indicating that it is a group of very high internal microdiversity (Additional file 1: Fig. S4) compared to other *Ca.* Patescibacteria represented by LAGs, which could explain their poor assembly results. Nevertheless, we used the four candidate bins to predict some of the ecological features of the microbes represented by them (Table 1).

**Table 1:** Properties of Baikalibacteria bins

Baikalibacteria Bin	Bin size (Mb)	GC content ± SD (%)	#Genes	Average gene size (bp)	Introns in 16S rRNA gene	Diversity-generating retroelements (DGRs)	Metagenomic recruitment at 95% (RPKG)	Metagenomic recruitment at 70% (RPKG)
RBin04	196.6	48.3 (± 2.5)	29,012	560.4	+	+ (1 sequence)	6.9	12.4
RBin08	10.7	38.6 (± 1.4)	15,847	551.7	+	–	8.7	14.9
RBin09	4.7	43.9 (± 2.1)	6544	599.7	+	–	12.8	20.5
RBin10	21.3	44.0 (± 2.0)	31,348	576.3	+	+ (2 sequences)	9.3	15.6

### Abundance and distribution in freshwater systems

**Fig. 3:**
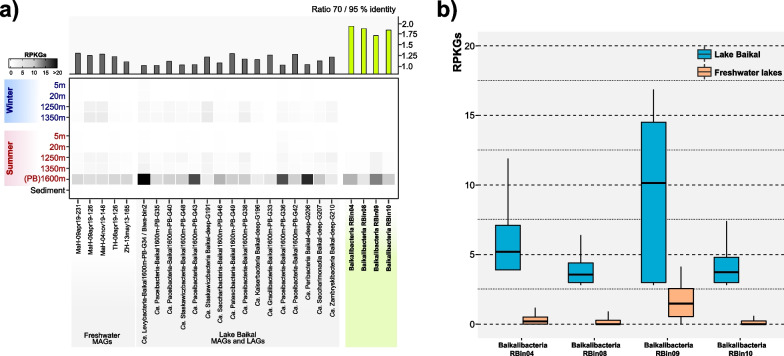
**a** Metagenomic recruitment along two depth profiles of Lake Baikal, collected during summer and winter seasons. Only MAGs retrieved from Lake Baikal and from a study that combined 17 different freshwater samples [12] that recruited at least 3 three reads per kilobase of genome per gigabase of metagenome (RPKGs) at 95% identity over 70% of their genome length in any of the samples are shown in the figure. The groups obtained from the binning of CCS5 reads (Baikalibacteria) are highlighted in green. **b** Boxplot of the metagenomic recruitment (in RPKGs, 95% identity) for each one of the CCS5 reads belonging to the four Baikalibacteria bins against two datasets: Lake Baikal metagenomes (blue boxes) and the compendium of 17 different freshwater samples (orange boxes) [12]. For each CCS5 read in a given dataset, only the highest recruitment value was taken into account. rRNA genes were masked for the recruitment

The16S rRNA sequences abundance of Baikalibacteria (149 out of 278 16S rRNA sequences affiliated with *Ca.* Patescibacteria) indicate that they could be by far the most abundant *Ca.* Patescibacteria in this habitat. Thus, we studied the recruitment of their bins at 95% identity (species level threshold) in all the available Baikal metagenomes and several other freshwater habitats. The depth profile in Baikal shows clearly how the novel group is absent in both surface and sediment samples (as were most other *Ca.* Patescibacteria MAGs and LAGs [11, 22], confirming that they are all inhabitants of the bathypelagic realm of the lake (Fig. 3A). Only some previously described MAGs obtained from the deep Baikal [11] recruited more than the Baikalibacteria bins. However, at 95% identity, the compounded Baikalibacteria bins recruited only 1.2% of the total number of Illumina reads from the 1350 m metagenome and 4.4% from the PacBio 1600 m metagenome. Likely, Bins 04, 08, 09, and 10 represent only a small part of the species-level diversity present in the new group. This could be expected due to their high diversity reflected by the ANIr (Additional file 1: Fig. S4), thus we carried out the recruitment at the genus (70% identity) (Fig. 3A, upper panel). At this identity Baikalibacteria bins recruit 8.3% of the 1600 m metagenome reads and 3.4% from the 1350 m metagenome showing them to be the dominant clade of *Ca.* Patescibacteria in the deep Lake Baikal and the most prevalent group after Actinobacteria.

In addition, none of the genomes downloaded from GenBank coming from similar environments such as aphotic groundwater systems recruited in Lake Baikal and vice versa (data not shown). Recently, a set of 119 metagenomic samples taken from 17 different freshwater lakes located in Europe and Asia described several *Ca.* Patescibacteria freshwater lineages [12]. Interestingly, there is a MAG found in Lake Biwa (Biwa-bin2) that shared a high nucleotide similarity (99.5%) to one from Lake Baikal, although samples were taken 3000 km apart. Therefore, we evaluated by metagenomic recruitment at 95% nucleotide identity whether the Baikalibacteria bins are present in these lakes. We evaluated each of the CCS reads as individuals, and for each of them, the best recruitment value (RPKG) in each dataset (10 and 119 metagenomes in Lake Baikal and the “freshwater” set, respectively) shows the apparent endemism of the Baikalibacteria group in Lake Baikal (Fig. 3B). Only RBin09 seemed to recruit significantly on a single dataset coming from Lake Thun [12], an alpine oligotrophic lake that seems to contain sequences related to RBin09 although at less than 95% similarity (Additional file 1: Fig. S5).

### Metabolism inferred from Baikalibacteria pangenomic bins

**Fig. 4:**
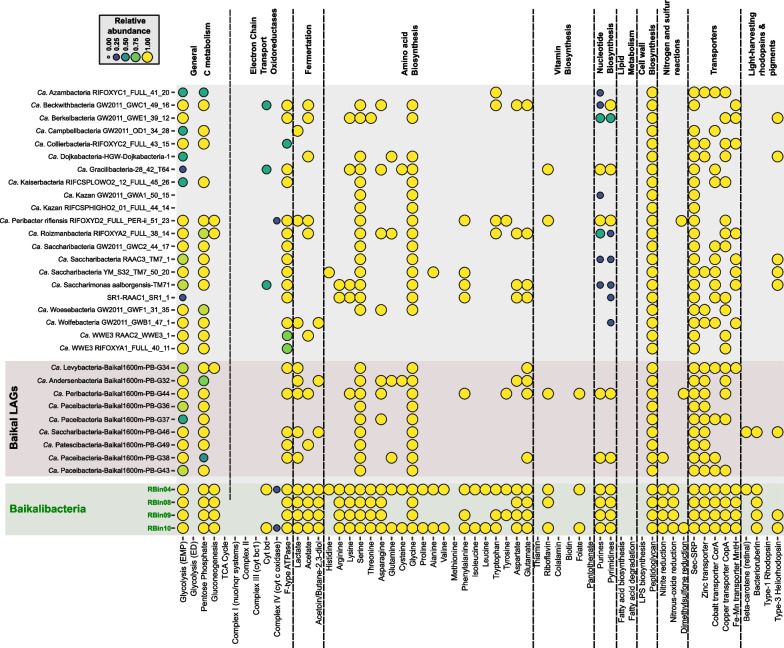
Inferred metabolism (KEGG) of the four Baikalibacteria bins (below) compared to LAGs (this work) and already published MAGs described as complete. The scale (color and size) indicates proportion of genes required for the full pathway detected

Metabolic inference of the Baikalibacteria pangenomes showed similar results to previously published studies carried out with MAGs from *Ca.* Patescibacteria from Baikal and elsewhere [8, 12, 20, 55]. Like the most complete MAGs [56], they lack most of the essential biosynthetic reactions to build nucleotides, amino acids, lipids, and vitamins (Fig. 4), making them highly dependent on outside sources. They also have in common the use of glucose to obtain energy via the Embden–Meyerhof–Parnas (EMP) pathway. However, although being found in oxic waters, all of them lack the oxidative pathways of the tricarboxylic acid cycle (TCA) and the electron chain transport complexes I to IV. Therefore, they likely rely on mixed-acid fermentative reactions of pyruvate to lactate, acetate, and Acetoin/Butane-2,3-diol to regenerate NAD^+^. It was also remarkable the abundance of Zn transporters among Baikal MAGs [11] and Baikalibacterial pangenomes, in contrast to their poor prevalence in other patescibacterial genomes. Like previously described MAGs from Lake Baikal superoxide dismutases of several kinds (Cu/Zn, Fe, or Mn) were found.

On the other hand, Baikalibacteria pangenomic bins had seem to have a more complex anabolism. For instance, they encode a substantial number of amino acid biosynthesis enzymes, many of which are not found in the isolate genomes and MAGs or Baikal LAGs (Fig. 4). However, these results should be taken with caution, since each bin represents an undetermined number of “closely related” cell genomes that can complement each other’s metabolism. Remarkably, we found within *Ca.* Patescibacteria Baikal-deep-G192, and the four Baikalibacteria bins, the genes (lycopene elongase (*lyeJ*), carotenoid 3,4-desaturase (*crt*D), and bisanhydrobacterioruberin hydratase (*cru*F)) required for the biosynthesis of the beta-carotene derivatives bacterioruberins, unusual carotenoids found in extremely halophilic archaea, and some Actinobacteria [57, 58]. In *Arthrobacter agilis* (Actinobacteria) or *Halobacterium salinarum* (Archaea) these three genes are forming a single gene cluster. However, in *Ca.* Saccharibacteria-Baikal1600m-PB-G46 *cru*F was detected far from *lye*J and *crt*D genes, and in the case of Baikalibacteria these genes were found on different CCS reads i.e. not forming a gene cluster or operon (Additional file 1: Fig. S6). The phylogenetic trees of the LyeJ and CrtD proteins also showed a polyphyletic relationship, where each bin forms an individual branch (Additional file 1: Fig. S6).

## Discussion

### Potential of long-read metagenomes

In this work, we have discovered a new and predominant group of *Ca.* Patescibacteria in an environment that was already studied in depth by short-read metagenome sequencing. They were missed before likely because of their abundance and enormous genomic diversity. Every method has biases and the assembly of MAGs from Illumina metagenomes has been shown to have many [23, 54, 59]. The use of third-generation sequencing alternatives that are not so reliant on assembly provides a way to bypass these biases and should be utilized when possible to advance in the discovery of prokaryotic diversity and to generate more reliable and informative MAGs (LAGs) [23, 59]. We have not been able to generate sizeable MAGs or LAGs for the new group, but by read binning, the study of the poorly assembled or even unassembled reads provides already a wealth of information about the biology of these microbes. The presence of the complete biosynthesis cluster of bacterioruberin deserves special attention. These C50 carotenoids detected in Actinobacteria have been associated with the adaptation of the membranes to low temperatures [57], which fits very well with the conditions of the deep lake Baikal that are at a constant 4 °C. The presence of special lipids (lysolipids) has been described before in CPR bacteria and its presence is justified by the requirements of high membrane curvature due to the small size [60]. This could be a role of bacterioruberins in *Ca.* Patescibacteria living in permanently cold deep Baikal waters. This characteristic links again *Ca.* Patescibacteria to Actinobacteria which appear to be the hosts for some of their groups [61–63].

### Are Baikalibacteria endemic of Lake Baikal?

Lake Baikal represents one of the most isolated and, in many ways, unique freshwater habitats on Earth. Due to its altitude (455 m) and remoteness (all major population centers are downstream), this environment has remained relatively pristine. It is also the largest and oldest lake on Earth (> 25 million years) and has several endemicities from sponges to seals [64]. Therefore, it would not be surprising to find endemic microbes. Although this is true for Baikalibacteria, there seems to be evidence of relatives being present, at least in small proportions and with less than species-level similarity in the deep alpine Lake Thun [12]. It has been found before that some bacteria from Lake Biwa (Japan) showed a very high similarity to MAGs of Baikal (> 99.5%). Thus, despite its remoteness, some Baikal microbial inhabitants have close relatives at great distances, which witness the amazing ubiquity of prokaryotic microbes.

## Concluding remarks

The *Ca.* Patescibacteria represent a major mystery of modern microbiology. They are surprisingly widespread and display a remarkable degree of sequence diversity. Whether this represents a long evolutionary time or a reflection of their lifestyle and molecular biology, cannot be discerned presently. Achieving axenic (monoclonal) cultures of these microbes has proven quite difficult and might be impossible unless the host is recognized and belongs to the rather restricted set that can be grown in the laboratory. The discovery of these dominant groups of CPRs in Lake Baikal opens a new gate to study their ecology and diversity, by culture or by metagenomics population genomics. The bathypelagic habitat of Lake Baikal is certainly special from many points of view (depth and temperature) but it is also homogenous (in time and space) and predictable (parameters change very little over seasons or years). The existence of the described diversified group indicates a complex species make up in a relatively homogeneous habitat seems to suggest that the reasons for their extreme genome diversity are not related to adaptation to deal with changes in time and/or space (of the CPR or their host). They could be more related to their molecular biology (mutation rates) or lifestyle like in the case of endosymbionts that have little selection pressure.

## Supplementary Information

**Additional file 1: Table S1.** Summary statistics of the Baikal 1600 m long-read sequencing and metagenomic assembly. **Fig. S1.** **A** Principal component analysis (PCA) between deep Lake Baikal metagenomes based on a Bray-Curtis similarity k-mer profile frequencies of sequencing reads. Red and blue dots represent summer and winter Illumina metagenomes, respectively, while the green dot is the sample retrieved in this study and sequenced with PacBio Sequel II. **B** Phylum-level composition based on 16S rRNA gene fragments (Illumina and PacBio CCS5 reads) of the different metagenomes. The single metagenome highlighted in green corresponds to the PacBio sequencing, whilst the rest of datasets belong to previous Illumina sequencing. The phylum Proteobacteria was divided into its class-level classification. Only those groups with abundance values larger than 1% in any of the metagenomes are shown. **C** Classification of the 1600 m PacBio CCS5 16S rRNA reads at a higher taxonomic resolution. Only sequences larger than 1000 nucleotides were considered. Sequences ascribed to the *Ca.* Patescibacteria phylum are highlighted in green. **Table S2.** Genomic parameters of LAGs recovered in this study. **Table S3.** Genomic parameters of LAGs recovered in this study with ANI > 99.5% to MAGs retrieved from Lake Baikal 1250 and 1350 m deep. **Fig. S2.** Alignment of two LAGs that are complete in a single contig and the respective MAG from the Illumina assembly. **Table S4.** Genomic parameters of the resulting bins from the Baikal 1600 m CCS sequences. The four Baikalibacteria bins are highlighted in yellow. **Fig. S3.** **A** Maximum likelihood phylogenetic tree of the Baikalibacteria 16S rRNA genes. Sequences outside the deep branch coming from Figure 1 were used as an outgroup for the tree. The reads from the four read bins are colored in the figure. **B** Diversity of 16S rRNA sequences of Baikalibacteria bins. Linear representation of selected CCS5 reads (indicated with a red circle in the left panel) containing a 16S rRNA gene. A pairwise blastn comparison among reads was performed to detect orthologous genes. **Fig. S4.** **A** Average nucleotide identity based on metagenomic reads (ANIr) of LAGs and the four Baikalibacteria Bins. **B** ANIr of ten randomly selected sequences of each Baikalibacteria bin. **Fig. S5.** Metagenomic recruitment of the largest fragment of Baikalibacteria RBin09 on Lake Thun 180 m deep. **Fig. S6.** Maximum likelihood phylogenetic tree of the **a** phytoene elongase (LyeJ), **b** carotenoid 3,4-desaturase (CrtD), and **c** bisanhydrobacterioruberin hydratase (CruF) proteins.

## Data Availability

Metagenomic datasets have been submitted to NCBI SRA and are available under BioProject accession number PRJNA924152 (Illumina reads: Sediment [SAMN32747376]; and PacBio CCS reads: 1600 m [SAMN32747378]). LAGs have been also uploaded to the BioProject accession number PRJNA924152.
