# Supplementary material for: A novel and diverse group of Candidatus Patescibacteria from bathypelagic Lake Baikal revealed through long-read metagenomics
Source: Environ Microbiome. 2023 Feb 23;18:12. doi: 10.1186/s40793-023-00473-1 (PMC9948471; doi:10.1186/s40793-023-00473-1)
Supplement: Supplementary file 1 — Additional file 1: Table S1. Summary statistics of the Baikal 1600 m long-read sequencing and metagenomic assembly. Fig. S1. A Principal component analysis (PCA) between deep Lake Baikal metagenomes based on a Bray-Curtis similarity k-mer profile frequencies of sequencing reads. Red and blue dots represent summer and winter Illumina metagenomes, respectively, while the green dot is the sample retrieved in this study and sequenced with PacBio Sequel II. B Phylum-level composition based on 16S rRNA gene fragments (Illumina and PacBio CCS5 reads) of the different metagenomes. The single metagenome highlighted in green corresponds to the PacBio sequencing, whilst the rest of datasets belong to previous Illumina sequencing. The phylum Proteobacteria was divided into its class-level classification. Only those groups with abundance values larger than 1% in any of the metagenomes are shown. C Classification of the 1600 m PacBio CCS5 16S rRNA reads at a higher taxonomic resolution. Only sequences larger than 1000 nucleotides were considered. Sequences ascribed to the Ca. Patescibacteria phylum are highlighted in green. Table S2. Genomic parameters of LAGs recovered in this study. Table S3. Genomic parameters of LAGs recovered in this study with ANI > 99.5% to MAGs retrieved from Lake Baikal 1250 and 1350 m deep. Fig. S2. Alignment of two LAGs that are complete in a single contig and the respective MAG from the Illumina assembly. Table S4. Genomic parameters of the resulting bins from the Baikal 1600 m CCS sequences. The four Baikalibacteria bins are highlighted in yellow. Fig. S3. A Maximum likelihood phylogenetic tree of the Baikalibacteria 16S rRNA genes. Sequences outside the deep branch coming from Figure 1 were used as an outgroup for the tree. The reads from the four read bins are colored in the figure. B Diversity of 16S rRNA sequences of Baikalibacteria bins. Linear representation of selected CCS5 reads (indicated with a red circle in the left panel) containing a 16S [file 40793_2023_473_MOESM1_ESM.pdf]

A novel and diverse group of Candidatus Patescibacteria from bathypelagic Lake Baikal revealed through long-read metagenomics

Jose M. Haro-Moreno[a], Pedro J. Cabello-Yeves[b], M. Pilar Garcillán-Barcia[c], Alexandra Zakharenko[d], Tamara I. Zemskaya[d], and Francisco Rodriguez-Valera[a,*]

[a]Evolutionary Genomics Group, Departamento Producción Vegetal y Microbiología, Universidad Miguel Hernández, Apartado 18, San Juan 03550, Alicante, Spain. [b]Cavanilles Institute of Biodiversity and Evolutionary Biology, University of Valencia, E-46980 Paterna, Valencia, Spain.[c]Instituto de Biomedicina y Biotecnología de Cantabria (IBBTEC), Universidad de Cantabria-Consejo Superior de Investigaciones Científicas, Santander, Spain. [d]Limnological Institute, Siberian Branch of the Russian Academy of Sciences, Irkutsk, Russia.

*Corresponding author: frvalera@umh.es

Evolutionary Genomics Group, Departamento Producción Vegetal y Microbiología, Universidad Miguel Hernández, Apartado 18, San Juan de Alicante, 03550 Alicante, Spain.

Phone +34-965919313, Fax +34-965 919457

SUPPORTING INFORMATION

Figure S1. A) Principal component analysis (PCA) between deep Lake Baikal metagenomes based on a Bray-Curtis similarity k-mer profile frequencies of sequencing reads. Red and blue dots represent summer and winter Illumina metagenomes, respectively, while the green dot is the sample retrieved in this study and sequenced with PacBio Sequel II. **B)** Phylum-level composition based on 16S rRNA gene fragments (Illumina and PacBio CCS5 reads) of the different metagenomes. The single metagenome highlighted in green corresponds to the PacBio sequencing, whilst the rest of datasets belong to previous Illumina sequencing. The phylum Proteobacteria was divided into its class-level classification. Only those groups with abundance values larger than 1 % in any of the metagenomes are shown. **C)** Classification of the 1600m PacBio CCS5 16S rRNA reads at a higher taxonomic resolution. Only sequences larger than 1,000 nucleotides were considered. Sequences ascribed to the *Ca.* Patescibacteria phylum are highlighted in green.

Figure S2. Alignment of two LAGs that are complete in a single contig and the respective MAG from the Illumina assembly.

Figure S3. A) Maximum likelihood phylogenetic tree of the Baikalibacteria 16S rRNA genes. Sequences outside the deep branch coming from Figure 1 were used as an outgroup for the tree. The reads from the four read bins are colored in the figure. **B)** Diversity of 16S rRNA sequences of Baikalibacteria bins. Linear representation of selected CCS5 reads (indicated with a red circle in the left panel) containing a 16S

rRNA gene. A pairwise blastn comparison among reads was performed to detect orthologous genes.

Figure S4. A) Average nucleotide identity based on metagenomic reads (ANIr) of LAGs and the four Baikalibacteria Bins. **B)** ANIr of ten randomly selected sequences of each Baikalibacteria bin.

Figure S5. Metagenomic recruitment of the largest fragment of Baikalibacteria RBin09 on Lake Thun 180 m deep.

Figure S6. Maximum likelihood phylogenetic tree of the a) phytoene elongase (LyeJ), b) carotenoid 3,4-desaturase (CrtD), and c) bisanhydrobacterioruberin hydratase (CruF) proteins.

Table S1. Summary statistics of the Baikal 1600 m long-read sequencing and metagenomic assembly.

Table S2. Genomic parameters of LAGs recovered in this study.

Table S3. Genomic parameters of LAGs recovered in this study with ANI > 99.5 % to MAGs retrieved from Lake Baikal 1250 and 1350 m deep.

Table S4. Genomic parameters of the resulting bins from the Baikal 1600 m CCS sequences. The four Baikalibacteria bins are highlighted in yellow.

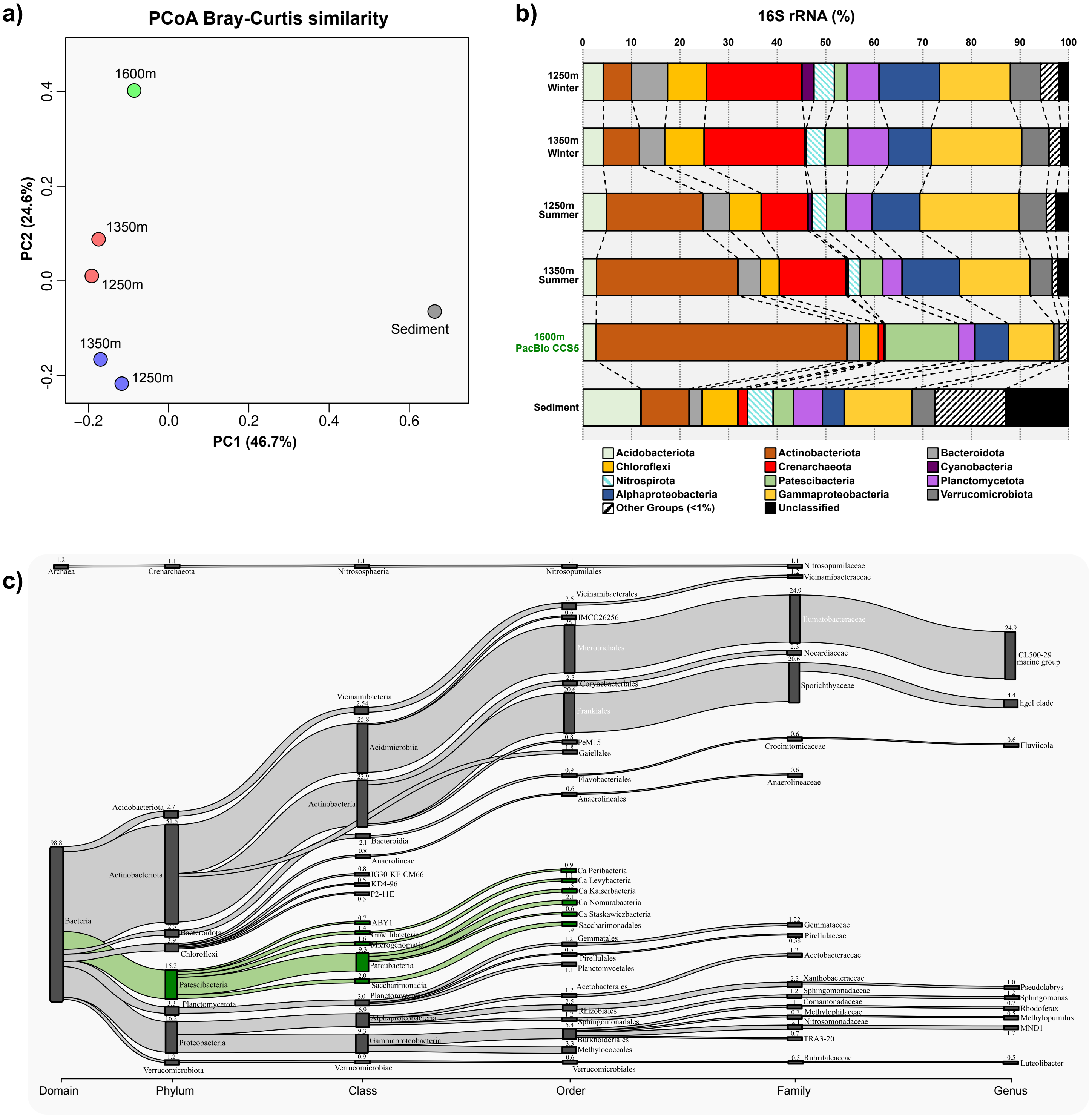

a)
PCoA Bray-Curtis similarity
PC2 (24.6%)
PC1 (46.7%)
1600m
1350m
1250m
Sediment
1350m
1250m
b)
16S rRNA (%)
1250m Winter
1350m Winter
1250m Summer
1350m Summer
1600m PacBio CCS5
Sediment
Acidobacteriota
Actinobacteriota
Bacteroidota
Chloroflexi
Crenarchaeota
Cyanobacteria
Nitrospirota
Patescibacteria
Planctomycetota
Alphaproteobacteria
Gammaproteobacteria
Verrucomicrobiota
Other Groups (<1%)
Unclassified
c)
Archaea
Crenarchaeota
Nitrososphaeria
Nitrosopumilales
Nitrosopumilaceae
Vicinamibacteraceae
Vicinamibacterales
IMCC26256
Ilumatobacteraceae
CL500-29 marine group
Microtrichales
Nocardiaceae
Corynebacteriales
Sporichthyaceae
hgcI clade
Vicinamibacteria
Frankiales
Acidimicrobiia
PeM15
Gaiellales
Crocinitomicaceae
Fluviicola
Flavobacteriales
Anaerolineaceae
Anaerolineales
Acidobacteriota
Actinobacteria
Bacteroidia
Anaerolineae
Actinobacteriota
JG30-KF-CM66
KD4-96
P2-11E
Bacteria
Ca Peribacteria
Ca Levybacteria
Ca Kaiserbacteria
Ca Nomurabacteria
Ca Staskawiczbacteria
ABY1
Saccharimonadales
Gracilibacteria
Gemmatales
Gemmataceae
Bacteroidota
Microgenomatia
Pirellulaceae
Chloroflexi
Pirellulales
Parcubacteria
Planctomycetales
Acetobacteraceae
Patescibacteria
Saccharimonadia
Acetobacterales
Xanthobacteraceae
Pseudolabrys
Planctomycetota
Sphingomonadaceae
Sphingomonas
Comamonadaceae
Rhodoferax
Rhizobiales
Methylophilaceae
Methylopumilus
Alphaproteobacteria
Sphingomonadales
Nitrosomonadaceae
MND1
Proteobacteria
Gammaproteobacteria
Burkholderiales
TRA3-20
Methylococcales
Verrucomicrobiota
Verrucomicrobiae
Verrucomicrobiales
Rubritaleaceae
Luteolibacter
Domain
Phylum
Class
Order
Family
Genus

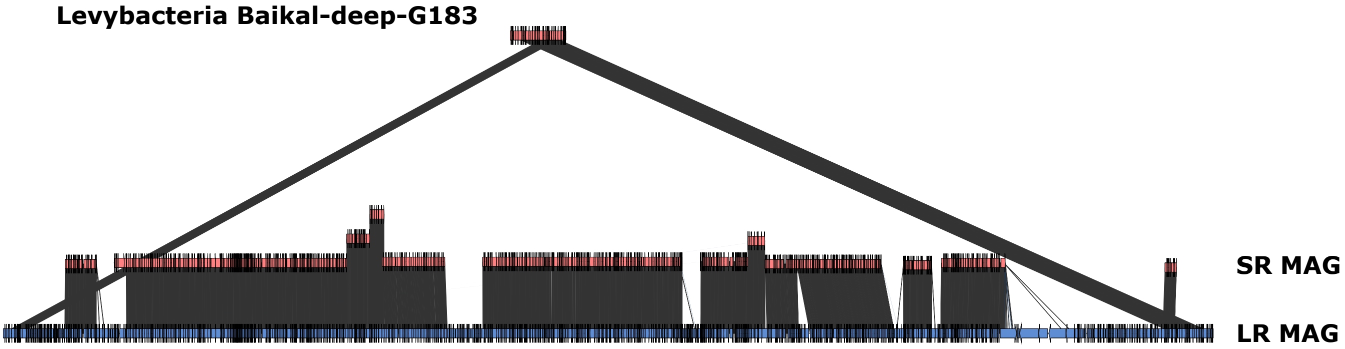
Levybacteria Baikal-deep-G183
SR MAG
LR MAG

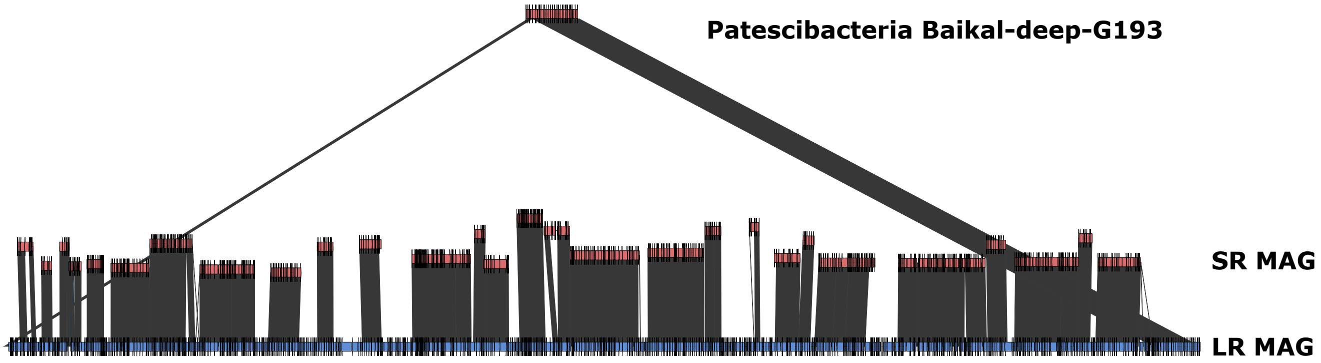
Patescibacteria Baikal-deep-G193
SR MAG
LR MAG

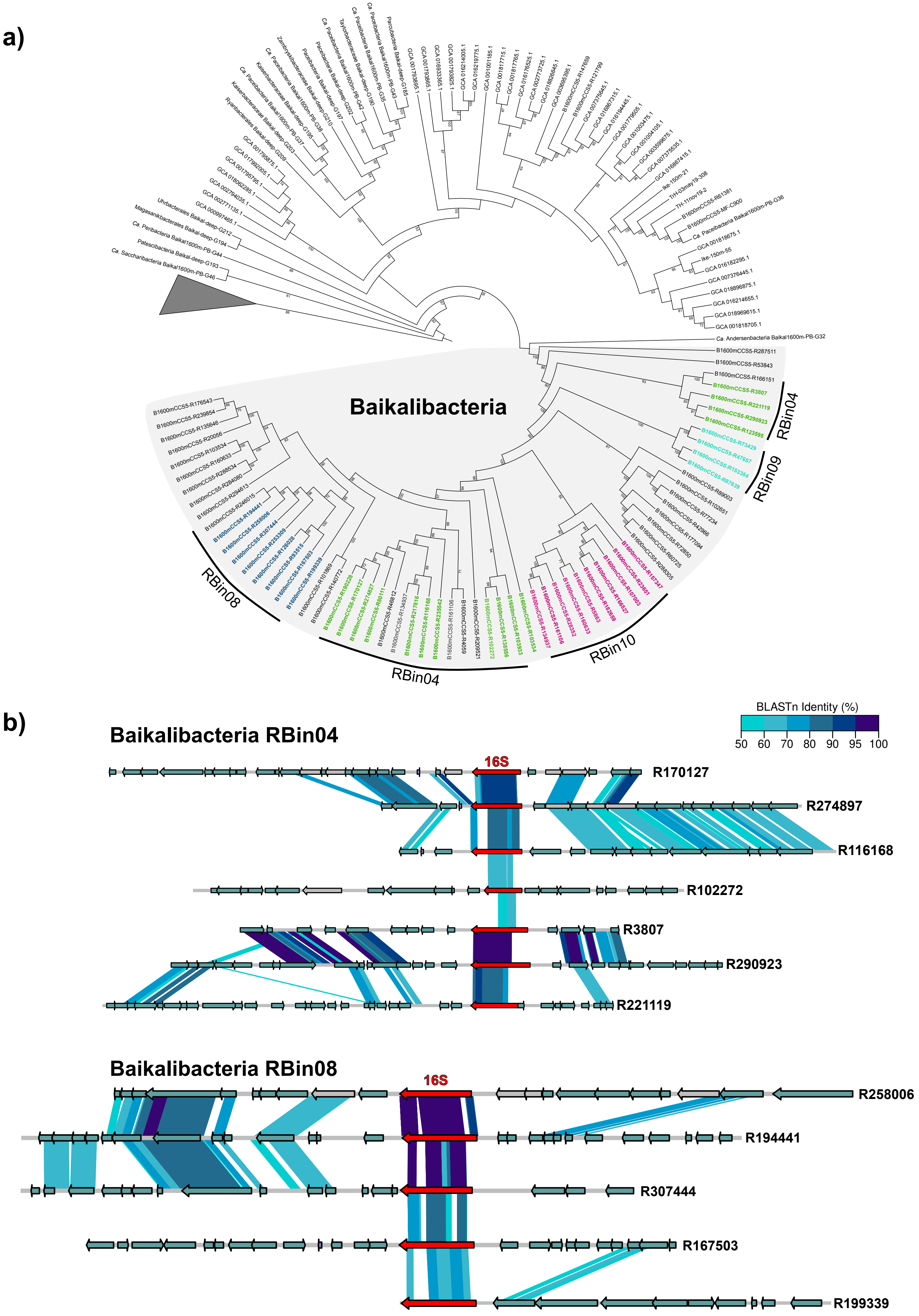
a)
Baikalibacteria
RBin04
RBin09
RBin10
RBin08
RBin04
b)
Baikalibacteria RBin04
BLASTn Identity (%)
50 60 70 80 90 95 100
16S
R170127
R274897
R116168
R102272
R3807
R290923
R221119
Baikalibacteria RBin08
16S
R258006
R194441
R307444
R167503
R199339

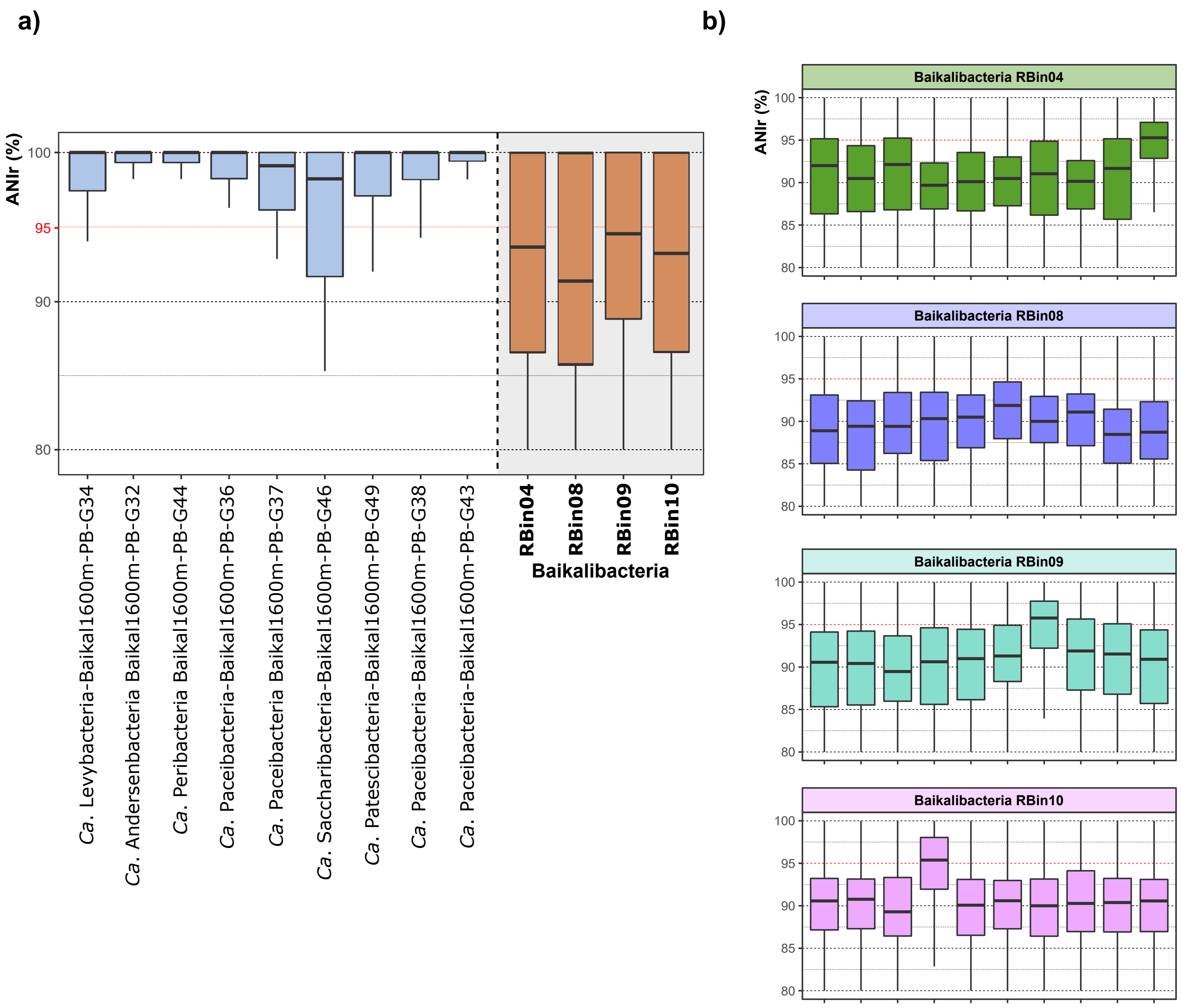
a)
b)
ANIr (%)
100
95
90
80
Ca. Levybacteria-Baikal1600m-PB-G34
Ca. Andersenbacteria Baikal1600m-PB-G32
Ca. Peribacteria Baikal1600m-PB-G44
Ca. Paceibacteria-Baikal1600m-PB-G36
Ca. Paceibacteria Baikal1600m-PB-G37
Ca. Saccharibacteria-Baikal1600m-PB-G46
Ca. Patescibacteria-Baikal1600m-PB-G49
Ca. Paceibacteria-Baikal1600m-PB-G38
Ca. Paceibacteria-Baikal1600m-PB-G43
RBin04
RBin08
RBin09
RBin10
Baikalibacteria
Baikalibacteria RBin04
Baikalibacteria RBin08
Baikalibacteria RBin09
Baikalibacteria RBin10
85

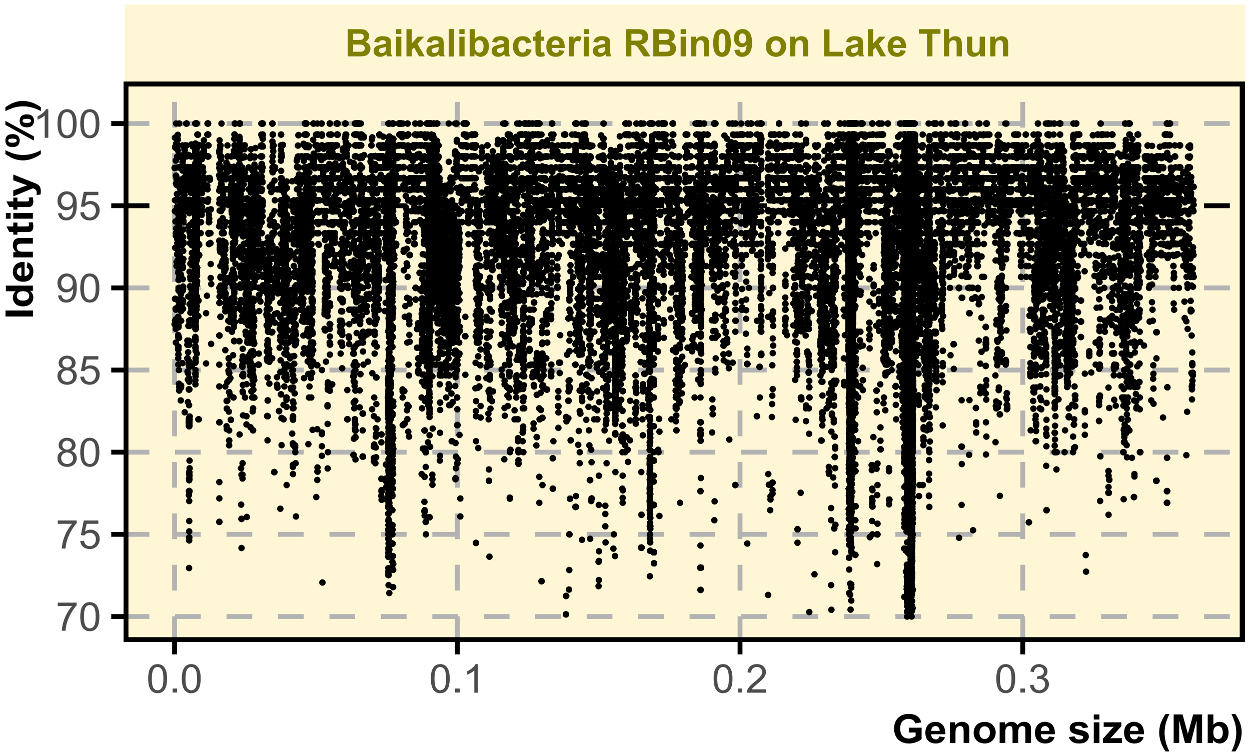
Baikalibacteria RBin09 on Lake Thun
Identity (%)
100
95
90
85
80
75
70
0.0
0.1
0.2
0.3
Genome size (Mb)

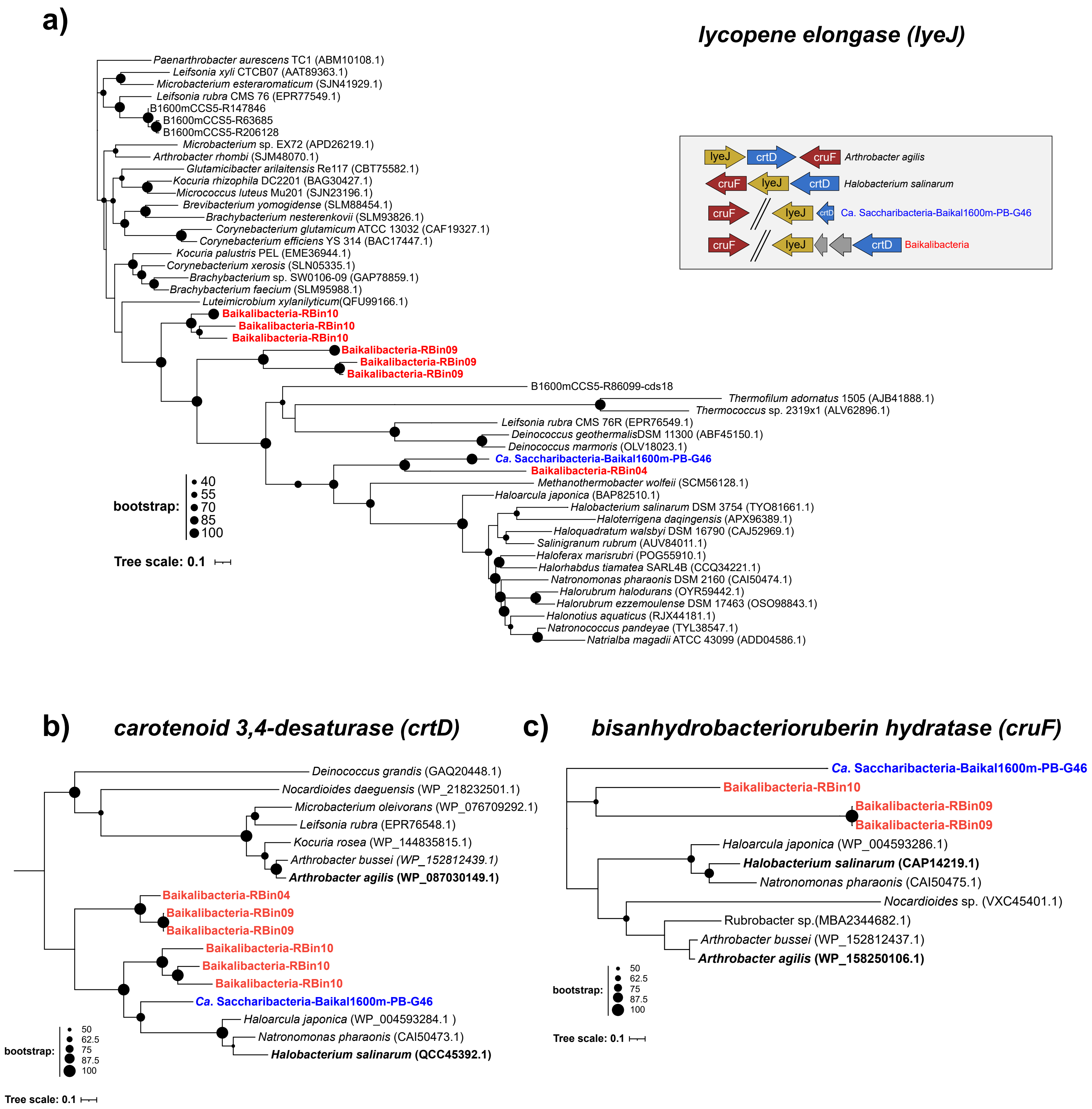
a)
lycopene elongase (lyeJ)
Paenarthrobacter aurescens TC1 (ABM10108.1)
Leifsonia xyli CTCB07 (AAT89363.1)
Microbacterium esteraromaticum (SJN41929.1)
Leifsonia rubra CMS 76 (EPR77549.1)
B1600mCCS5-R147846
B1600mCCS5-R63685
B1600mCCS5-R206128
Microbacterium sp. EX72 (APD26219.1)
Arthrobacter rhombi (SJM48070.1)
Glutamicibacter arilaitensis Re117 (CBT75582.1)
Kocuria rhizophila DC2201 (BAG30427.1)
Micrococcus luteus Mu201 (SJN23196.1)
Brevibacterium yomogidense (SLM88454.1)
Brachybacterium nesterenkovii (SLM93826.1)
Corynebacterium glutamicum ATCC 13032 (CAF19327.1)
Corynebacterium efficiens YS 314 (BAC17447.1)
Kocuria palustris PEL (EME36944.1)
Corynebacterium xerosis (SLN05335.1)
Brachybacterium sp. SW0106-09 (GAP78859.1)
Brachybacterium faecium (SLM95988.1)
Luteimicrobium xylanilyticum(QFU99166.1)
Baikalibacteria-RBin10
Baikalibacteria-RBin10
Baikalibacteria-RBin10
Baikalibacteria-RBin09
Baikalibacteria-RBin09
Baikalibacteria-RBin09
B1600mCCS5-R86099-cds18
Thermofilum adornatus 1505 (AJB41888.1)
Thermococcus sp. 2319x1 (ALV62896.1)
Leifsonia rubra CMS 76R (EPR76549.1)
Deinococcus geothermalisDSM 11300 (ABF45150.1)
Deinococcus marmoris (OLV18023.1)
Ca. Saccharibacteria-Baikal1600m-PB-G46
Baikalibacteria-RBin04
Methanothermobacter wolfeii (SCM56128.1)
Haloarcula japonica (BAP82510.1)
Halobacterium salinarum DSM 3754 (TYO81661.1)
Haloterrigena daqingensis (APX96389.1)
Haloquadratum walsbyi DSM 16790 (CAJ52969.1)
Salinigranum rubrum (AUV84011.1)
Haloferax marisrubri (POG55910.1)
Halorhabdus tiamatea SARL4B (CCQ34221.1)
Natronomonas pharaonis DSM 2160 (CAI50474.1)
Halorubrum halodurans (OYR59442.1)
Halorubrum ezzemoulense DSM 17463 (OSO98843.1)
Halonotius aquaticus (RJX44181.1)
Natronococcus pandeyae (TYL38547.1)
Natrialba magadii ATCC 43099 (ADD04586.1)
bootstrap:
40
55
70
85
100
Tree scale: 0.1
lyeJ crtD cruF Arthrobacter agilis
cruF lyeJ crtD Halobacterium salinarum
cruF lyeJ crtD Ca. Saccharibacteria-Baikal1600m-PB-G46
cruF lyeJ crtD Baikalibacteria
b)
carotenoid 3,4-desaturase (crtD)
Deinococcus grandis (GAQ20448.1)
Nocardioides daeguensis (WP_218232501.1)
Microbacterium oleivorans (WP_076709292.1)
Leifsonia rubra (EPR76548.1)
Kocuria rosea (WP_144835815.1)
Arthrobacter bussei (WP_152812439.1)
Arthrobacter agilis (WP_087030149.1)
Baikalibacteria-RBin04
Baikalibacteria-RBin09
Baikalibacteria-RBin09
Baikalibacteria-RBin10
Baikalibacteria-RBin10
Baikalibacteria-RBin10
Ca. Saccharibacteria-Baikal1600m-PB-G46
Haloarcula japonica (WP_004593284.1)
Natronomonas pharaonis (CAI50473.1)
Halobacterium salinarum (QCC45392.1)
bootstrap:
50
62.5
75
87.5
100
Tree scale: 0.1
c)
bisanhydrobacterioruberin hydratase (cruF)
Ca. Saccharibacteria-Baikal1600m-PB-G46
Baikalibacteria-RBin10
Baikalibacteria-RBin09
Baikalibacteria-RBin09
Haloarcula japonica (WP_004593286.1)
Halobacterium salinarum (CAP14219.1)
Natronomonas pharaonis (CAI50475.1)
Nocardioides sp. (VXC45401.1)
Rubrobacter sp.(MBA2344682.1)
Arthrobacter bussei (WP_152812437.1)
Arthrobacter agilis (WP_158250106.1)
bootstrap:
50
62.5
75
87.5
100
Tree scale: 0.1

Table S1. Summary statistics of the Baikal 1600 m long-read sequencing and metagenomic assembly.

	1600 m deep PacBio Sequel II	
**Read Type (Proccesing)**	**Raw Reads**	**CCS Reads 5**
**Sequencing statistics (Reads > 1 Kb):**		
#Sequences (Millions)	6.61	0.31
Nucleotides sequenced (Gb)	90.83	4.46
Largest read size (bp)	435,307	29,377
Average length read size (bp)	13,744	14,334
**Predicted proteins (Reads > 1 Kb):**		
Proteins (Millions)	96.0	6.6
Average Protein Size (aa)	135.4	183.4
Proteins / Mb Sequenced	1,057.0	1,482.4
**Assembly statistics (Contigs > 1 Kb):**		
#Sequences	-	2,913
#Nucleotides assembled (Mb)	-	192.2
Largest contig size (bp)	-	2,256,336
Average length contig size (bp)	-	65,968

Table S2. Genomic parameters of LAGs recovered in this study.

Group	Genome Name	Match to Lake Baikal Illumina MAG (>99% ANI)	cMAG	Genome Size (bp)	Number Contigs	Longest Contig Size(bp)	Shortest Contig Size(bp)	GC (%)	Number Proteins	Average Protein Size	Median Intergenic spacer (bp)	Coding Density (%)	Completeness (%)	Contamination (%)	GTDB Taxonomy	GTDB Classification Method
Acidobacteria	Vicinamibacterales-Baikal1600m-PB-G1	Acidobacteria Baikal-deep-G12 / Baikal-deep-G4	--	4,886,496	43	733,256	2,283	65.3	4,622		43	90	92.35	4.84	d__Bacteria;p__Acidobacteriota;c__Vicinamibacteria;o__Vicinamibacterales;f__UBA2999;g__12-FULL-67-14b;s__	topology and ANI
Actinobacteria	Acidimicrobiia-Baikal1600m-PB-G2	Acidimicrobia Baikal-deep-G23	--	2,144,435	8	1,670,992	6,563	58.3	2,139	308.8	31	93	77.8	1.3	d__Bacteria;p__Actinobacteriota;c__Acidimicrobiia;o__IMCC26256;f__IMCC26256;g__;s__	topology and ANI
Actinobacteria	Acidimicrobiia-Baikal1600m-PB-G3	Baikal-G2 / Acidimicrobiia Baikal-deep-G22	--	2,083,303	11	823,156	19,788	53.3	2,263	281.7	25.5	93	96.6	2.7	d__Bacteria;p__Actinobacteriota;c__Acidimicrobiia;o__IMCC26256;f__PALSA-555;g__;s__	topology and ANI
Actinobacteria	Ca. Nanopelagicus-Baikal1600m-PB-G4	Ca. Nanopelagicales Baikal-deep-G27	--	1,215,619	6	725,322	3,126	42.1	1,263	300.7	15	95	79.8	0.0	d__Bacteria;p__Actinobacteriota;c__Actinomycetia;o__Nanopelagicales;f__Nanopelagicaceae;g__Nanopelagicus;s__	topology and ANI
Actinobacteria	Ca. Planktophila-Baikal1600m-PB-G5	Ca. Planktophila Baikal-deep-G30	--	1,448,997	10	388,832	15,501	48.1	1,496	303.1	12	95	81.2	0.5	d__Bacteria;p__Actinobacteriota;c__Actinomycetia;o__Nanopelagicales;f__Nanopelagicaceae;g__Planktophila;s__	topology and ANI
Actinobacteria	Ca. Planktophila-Baikal1600m-PB-G6		--	1,101,041	6	389,753	47,718	46.1	1,519	208.2	26	87	59.7	0.3	d__Bacteria;p__Actinobacteriota;c__Actinomycetia;o__Nanopelagicales;f__Nanopelagicaceae;g__Planktophila;s__	topology and ANI
Actinobacteria	Ca. Planktophila-Baikal1600m-PB-G7		--	1,302,135	7	414,753	11,918	47.5	1,513	257.3	20	91	74.7	1.1	d__Bacteria;p__Actinobacteriota;c__Actinomycetia;o__Nanopelagicales;f__Nanopelagicaceae;g__Planktophila;s__	topology and ANI
Actinobacteria	Ilumatobacteraceae-Baikal1600m-PB-G8		--	1,440,491	15	284,918	8,373	52.0	1,641	274.6	16	94	72.2	0.9	d__Bacteria;p__Actinobacteriota;c__Acidimicrobiia;o__Acidimicrobiales;f__Ilumatobacteraceae;g__UBA3006;s__	topology and ANI
Actinobacteria	Ilumatobacteraceae-Baikal1600m-PB-G9		--	2,010,927	17	443,986	8,749	47.7	2,078	302.0	20	94	86.4	4.7	d__Bacteria;p__Actinobacteriota;c__Acidimicrobiia;o__Acidimicrobiales;f__Ilumatobacteraceae;g__BACL27;s__	topology and ANI
Actinobacteria	Ilumatobacteraceae-Baikal1600m-PB-G10	Ilumatobacteraceae Baikal-deep-G17	--	2,120,470	3	1,629,087	59,052	51.1	2,106	319.0	17	96	97.9	1.3	d__Bacteria;p__Actinobacteriota;c__Acidimicrobiia;o__Acidimicrobiales;f__Ilumatobacteraceae;g__UBA2093;s__	topology and ANI
Actinobacteria	Ilumatobacteraceae-Baikal1600m-PB-G11		--	2,183,118	7	814,896	20,138	51.2	2,192	311.7	18	95	97.9	1.3	d__Bacteria;p__Actinobacteriota;c__Acidimicrobiia;o__Acidimicrobiales;f__Ilumatobacteraceae;g__UBA2093;s__	topology and ANI
Actinobacteria	Nanopelagicales-Baikal1600m-PB-G12	Ca. Nanopelagicales Baikal-deep-G14	--	2,345,733	11	855,656	9,998	57.8	2,432	292.3	28	92	94.7	0.5	d__Bacteria;p__Actinobacteriota;c__Actinomycetia;o__Nanopelagicales;f__S36-B12;g__GCA-2737125;s__	topology and ANI
Actinobacteria	Nanopelagicales-Baikal1600m-PB-G13		--	1,654,748	7	990,038	9,697	46.4	1,743	290.7	21	93	84.2	0.0	d__Bacteria;p__Actinobacteriota;c__Actinomycetia;o__Nanopelagicales;f__Nanopelagicaceae;g__QYPT01;s__	topology and ANI
Actinobacteria	Nanopelagicales-Baikal1600m-PB-G14		--	1,131,846	12	238,295	23,381	44.1	1,261	276.3	17	93	66.5	4.7	d__Bacteria;p__Actinobacteriota;c__Actinomycetia;o__Nanopelagicales;f__Nanopelagicaceae;g__UBA7398;s__	topology and ANI
Actinobacteria	Thermoleophilia-Baikal1600m-PB-G15	Thermoleophilia Baikal-deep-G28	--	1,630,020	3	840,662	119,520	68.1	1,716	270.9	39	86	89.9	0.9	d__Bacteria;p__Actinobacteriota;c__Thermoleophilia;o__Ga0077560;f__Ga0077560;g__SYFI01;s__	topology and ANI
Actinobacteria	Thermoleophilia-Baikal1600m-PB-G16	Thermoleophilia Baikal-deep-G21	**Yes**	2,256,336	1	--	--	63.5	2,266	309.1	22	94	98.4	0.4	d__Bacteria;p__Actinobacteriota;c__Thermoleophilia;o__Gaiellales;f__F1-60-MAGs149;g__F1-60-MAGs149;s__	topology and ANI
Actinobacteria	Williamsia-Baikal1600m-PB-G17		--	6,364,282	19	1,994,645	24,875	64.7	6,233	301.8	55	89	98.2	1.3	d__Bacteria;p__Actinobacteriota;c__Actinomycetia;o__Mycobacteriales;f__Mycobacteriaceae;g__Williamsia;s__Williamsia muralis	topology and ANI
Alphaproteobacteria	Acetobacteraceae-Baikal1600m-PB-G18		--	2,750,257	31	247,627	16,830	60.6	3,207	230.1	71	81	67.07	0.83	d__Bacteria;p__Proteobacteria;c__Alphaproteobacteria;o__Acetobacterales;f__Acetobacteraceae;g__;s__	topology
Alphaproteobacteria	Acetobacteraceae-Baikal1600m-PB-G19	Acetobacteraceae Baikal-deep-G58	--	3,427,791	9	1,497,302	25,026	61.6	3,310	295.7	61	86	98.51	1.99	d__Bacteria;p__Proteobacteria;c__Alphaproteobacteria;o__Acetobacterales;f__Acetobacteraceae;g__;s__	topology
Alphaproteobacteria	Ca. Fonsibacter-Baikal1600m-PB-G20	Ca. Fonsibacter Baikal-deep-G36	--	946,322	4	627,042	54,036	29.1	1,069	277.9	4	95	75.47	0	d__Bacteria;p__Proteobacteria;c__Alphaproteobacteria;o__Pelagibacterales;f__Pelagibacteraceae;g__Fonsibacter;s__	topology and ANI
Alphaproteobacteria	Pseudolabrys-Baikal1600m-PB-G21	Pseudolabrys Baikal-deep-G38 / Baikal-deep-G61	--	2,738,833	15	461,811	9,688	62.1	2,796	287.5	59	89	86.33	0.79	d__Bacteria;p__Proteobacteria;c__Alphaproteobacteria;o__Rhizobiales;f__Xanthobacteraceae;g__Pseudolabrys;s__	topology and ANI
Alphaproteobacteria	Reyranella-Baikal1600m-PB-G22		--	5,073,546	16	1,529,917	90,289	65.7	5,472	275.6	32	90	84.93	4.96	d__Bacteria;p__Proteobacteria;c__Alphaproteobacteria;o__Reyranellales;f__Reyranellaceae;g__Reyranella;s__	topology and ANI
Alphaproteobacteria	Xanthobacteraceae-Baikal1600m-PB-G23		--	4,847,097	30	780,725	20,618	64.8	4,935	284.4	68	87	95.28	2.69	d__Bacteria;p__Proteobacteria;c__Alphaproteobacteria;o__Rhizobiales;f__Xanthobacteraceae;g__Z2-YC6860;s__	topology and ANI
Bacteroidetes	Bacteroidetes-Baikal1600m-PB-G24	Bacteroidetes Baikal-deep-G73	--	1,675,431	10	367,283	40,083	31.5	1,619	325.1	11	95	87.7	0	d__Bacteria;p__Bacteroidota;c__UBA10030;o__UBA10030;f__UBA8401;g__SXSI01;s__SXSI01 sp005792285	topology and ANI
Bacteroidetes	Crocinitomicaceae-Baikal1600m-PB-G25		--	1,234,646	14	363,284	40,314	34.0	1,316.0	280.9	34	90	51.77	1.62	d__Bacteria;p__Bacteroidota;c__Bacteroidia;o__Flavobacteriales;f__Crocinitomicaceae;g__UBA952;s__	topology and ANI
Bacteroidetes	Chitinophagaceae-Baikal1600m-PB-G26		--	1,885,910	2	1,086,709	799,201	35.0	1,790	324.6	44	93	95.44	0.49	d__Bacteria;p__Bacteroidota;c__Bacteroidia;o__Chitinophagales;f__Chitinophagaceae;g__VBAS01;s__VBAS01 sp004322425	topology and ANI
Chloroflexi	Anaerolineales-Baikal1600m-PB-G27		--	1,315,299	11	486,992	40,178	62.2	1,283	303.7	65	89	50.3	1.82	d__Bacteria;p__Chloroflexota;c__Anaerolineae;o__Anaerolineales;f__UBA11657;g__BD1;s__	taxonomic novelty determined using RED
Betaproteobacteria	Burkholderiales-Baikal1600m-PB-G28		--	3,381,930	41	247,617	10,406	59.6	3,695	254.6	80	84	50.3	0.83	d__Bacteria;p__Proteobacteria;c__Gammaproteobacteria;o__Burkholderiales;f__SG8-41;g__;s__	taxonomic novelty determined using RED
Betaproteobacteria	Burkholderiales-Baikal1600m-PB-G29	Betaproteobacteria Baikal-deep-G89	--	2,746,740	11	863,158	15,539	63.0	2,865	293.1	24	92	96.76	2.78	d__Bacteria;p__Proteobacteria;c__Gammaproteobacteria;o__Burkholderiales;f__SG8-39;g__RBG-16-66-20;s__	topology and ANI
Gammaproteobacteria	Methyloglobulus-Baikal1600m-PB-G30	Methyloglobulus Baikal-deep-G142	--	2,053,671	12	506,236	36,921	45.7	2,056	278.2	87	85	95.26	1.27	d__Bacteria;p__Proteobacteria;c__Gammaproteobacteria;o__Methylococcales;f__Methylomonadaceae;g__Methyloglobulus;s__	topology and ANI
Betaproteobacteria	Methylopumilus-Baikal1600m-PB-G31	Methylopumilus planktonicus Baikal-deep-G83	--	1,302,697	3	641,110	298,451	35.8	1,403	289.1	15	94	95.6	0	d__Bacteria;p__Proteobacteria;c__Gammaproteobacteria;o__Burkholderiales;f__Methylophilaceae;g__Methylopumilus;s__	topology and ANI
Patescibacteria	Ca. Andersenbacteria-Baikal1600m-PB-G32		**Yes**	962,557	1	962,557	962,557	43.7	1,047	277.7	20	92	100.0*	0.0*	d__Bacteria;p__Patescibacteria;c__Andersenbacteria;o__UBA10190;f__UBA10190;g__HO2-45-11b;s__	taxonomic classification defined by topology and ANI
Patescibacteria	Ca. Gracilibacteria-Baikal1600m-PB-G33	Paceibacteria Baikal-deep-G197	--	919,098	17	231,563	6,913	44.3	1,000	263.7	49	87	67.4*	4.98*	d__Bacteria;p__Patescibacteria;c__Gracilibacteria;o__UBA1369;f__UBA1369;g__PALSA-1335;s__	taxonomic classification defined by topology and ANI
Patescibacteria	Ca. Levybacteria-Baikal1600m-PB-G34	Levybacteria Baikal-deep-G183	**Yes**	907,563	1	907,563	907,563	32.0	846	323.8	39	92	97.7*	0.0*	d__Bacteria;p__Patescibacteria;c__Microgenomatia;o__Levybacterales;f__UBA12049;g__;s__	taxonomic classification fully defined by topology
Patescibacteria	Ca. Paceibacteria-Baikal1600m-PB-G35	Parcubacteria Baikal-deep-G184	--	1,059,132	6	755,885	3,509	40.7	1,097	270.1	75	85	93.0*	0.0*	d__Bacteria;p__Patescibacteria;c__Paceibacteria;o__UBA9983;f__UBA11359_A;g__;s__	taxonomic classification defined by topology and ANI
Patescibacteria	Ca. Paceibacteria-Baikal1600m-PB-G36	Paceibacteria Baikal-deep-G200	**Yes**	582,052	1	582,052	582,052	49.5	651	260.8	45	89	97.7*	0.0*	d__Bacteria;p__Patescibacteria;c__Paceibacteria;o__UBA6257;f__UBA11618;g__;s__	taxonomic classification defined by topology and ANI
Patescibacteria	Ca. Paceibacteria-Baikal1600m-PB-G37		**Yes**	583,336	1	583,336	583,336	49.3	635	270.3	27	90	97.7*	0.0*	d__Bacteria;p__Patescibacteria;c__Paceibacteria;o__UBA9983;f__UBA2163;g__C7867-001;s__	taxonomic classification defined by topology and ANI
Patescibacteria	Ca. Paceibacteria-Baikal1600m-PB-G38	Kaiserbacteraceae Baikal-deep-G195	**Yes**	803,329	1	803,329	803,329	53.5	882	265.1	42	89	97.7*	0.0*	d__Bacteria;p__Patescibacteria;c__Paceibacteria;o__UBA9983;f__UBA2163;g__UBA10103;s__	taxonomic classification defined by topology and ANI
Patescibacteria	Ca. Paceibacteria-Baikal1600m-PB-G39		--	464,636	5	205,777	54,579	35.5	563	233.3	47	87	62.8*	0.0*	d__Bacteria;p__Patescibacteria;c__Paceibacteria;o__UBA9983;f__UBA9973;g__UBA9973;s__	taxonomic classification defined by topology and ANI
Patescibacteria	Ca. Paceibacteria-Baikal1600m-PB-G40	Parcubacteria-Baikal-deep-G185	--	592,655	5	171,467	16,160	38.3	617	254.6	103	81	74.4*	0.0*	d__Bacteria;p__Patescibacteria;c__Paceibacteria;o__UBA9983;f__Zambryskibacteraceae;g__C7867-006;s__	taxonomic classification defined by topology and ANI
Patescibacteria	Ca. Paceibacteria-Baikal1600m-PB-G41		--	634,378	4	380,661	64,066	37.7	768	225.8	65	83	100.0*	0.0*	d__Bacteria;p__Patescibacteria;c__Paceibacteria;o__UBA9983;f__UBA5272;g__UBA11704;s__	taxonomic classification defined by topology and ANI
Patescibacteria	Ca. Paceibacteria-Baikal1600m-PB-G42	Paceibacteria Baikal-deep-G202	--	558,371	2	485,322	73,049	39.4	639	249.3	46	87	86.1*	0.0*	d__Bacteria;p__Patescibacteria;c__Paceibacteria;o__UBA9983;f__Zambryskibacteraceae;g__;s__	taxonomic classification fully defined by topology
Patescibacteria	Ca. Paceibacteria-Baikal1600m-PB-G43	Taylorbacteraceae Baikal-deep-G189	**Yes**	754,737	1	754,737	754,737	39.2	744	308.2	27	92	97.7*	2.3*	d__Bacteria;p__Patescibacteria;c__Paceibacteria;o__UBA9983;f__UBA11359;g__SCTV01;s__	taxonomic classification defined by topology and ANI
Patescibacteria	Ca. Peribacteria-Baikal1600m-PB-G44		**Yes**	1,119,680	1	1,119,680	1,119,680	51.7	1,045	325.1	43	92	100.0*	0.0*	d__Bacteria;p__Patescibacteria;c__Gracilibacteria;o__Peribacterales;f__Peribacteraceae;g__2-02-FULL-51-10;s__	taxonomic classification defined by topology and ANI
Patescibacteria	Ca. Saccharibacteria-Baikal1600m-PB-G45		--	571,293	7	142,366	40,134	40.6	670	243.6	45	87	86.1*	0.0*	d__Bacteria;p__Patescibacteria;c__Saccharimonadia;o__Saccharimonadales;f__AWTP1-31;g__;s__	taxonomic classification defined by topology and ANI
Patescibacteria	Ca. Saccharibacteria-Baikal1600m-PB-G46	Patescibacteria Baikal-deep-G192	**Yes**	724,546	1	724,546	724,546	46.2	733	282.9	48	87	100.0*	0.0*	d__Bacteria;p__Patescibacteria;c__Saccharimonadia;o__;f__;g__;s__	taxonomic novelty determined using RED
Patescibacteria	Ca. Saccharibacteria-Baikal1600m-PB-G47		--	699,520	8	116,516	35,645	45.3	887	225.0	47	87	67.4*	0.0*	d__Bacteria;p__Patescibacteria;c__Saccharimonadia;o__Saccharimonadales;f__Saccharimonadaceae;g__2011-GWC2-44-17;s__	ANI
Patescibacteria	Ca. Staskawiczbacteria-Baikal1600m-PB-G48	Staskawiczbacteriaceae Baikal-deep-G187	--	522,042	2	500,723	21,319	38.3	599	248.0	37	87	90.7*	0.0*	d__Bacteria;p__Patescibacteria;c__Paceibacteria;o__Paceibacterales;f__Staskawiczbacteraceae;g__01-FULL-38-12b;s__	taxonomic classification defined by topology and ANI
Patescibacteria	Patescibacteria-Baikal1600m-PB-G49	Patescibacteria Baikal-deep-G193	**Yes**	737,753	1	737,753	737,753	53.7	833	262.5	41	90	97.7*	0.0*	d__Bacteria;p__Patescibacteria;c__;o__;f__;g__;s__	taxonomic novelty determined using RED
Planctomycetes	Gemmataceae-Baikal1600m-PB-G50	Gemmataceae Baikal-deep-G161	--	4,600,205	10	1,108,979	41,480	55.5	4,068	337.6	61	90	97.24	1.14	d__Bacteria;p__Planctomycetota;c__Planctomycetes;o__Gemmatales;f__Gemmataceae;g__;s__	taxonomic novelty determined using RED
Planctomycetes	Gemmataceae-Baikal1600m-PB-G51	Gemmataceae Baikal-deep-G173	--	4,632,407	21	804,744	45,209	52.8	3,988	336.7	72	88	93.18	1.14	d__Bacteria;p__Planctomycetota;c__Planctomycetes;o__Gemmatales;f__Gemmataceae;g__SYLG01;s__	topology and ANI
Planctomycetes	Planctomycetales-Baikal1600m-PB-G52		--	6,369,109	75	432,859	3,160	59.6	5,345	348.8	92	89	77.96	2.25	d__Bacteria;p__Planctomycetota;c__Planctomycetes;o__Planctomycetales;f__Planctomycetaceae;g__SXKJ01;s__	topology and ANI
Planctomycetes	Zavarzinella-Baikal1600m-PB-G53	Gemmataceae Baikal-deep-G171	--	5,219,492	29	457,339	54,645	58.5	4,826	307.1	93	86	75.29	4.55	d__Bacteria;p__Planctomycetota;c__Planctomycetes;o__Gemmatales;f__Gemmataceae;g__Zavarzinella;s__	topology and ANI
Thaumarchaeota	Nitrosarchaeum-Baikal1600m-PB-G54	Baikal-G1 / Nitrosarchaeum Baikal-deep-G181	--	1,280,083	4	793,988	9,722	32.4	1,554	242.7	43	89	99.03	2.43	chaea;p__Thermoproteota;c__Nitrososphaeria;o__Nitrososphaerales;f__Nitrosopumilaceae;g__Nitrosarchaeum;s__Nitrosarchaeum sp0027	topology and ANI
Thaumarchaeota	Nitrosarchaeum-Baikal1600m-PB-G55	Nitrosarchaeum Baikal-deep-G180	--	1,245,765	2	764,480	481,285	33.3	1,520	243.6	44	90	99.03	0.97	d__Archaea;p__Thermoproteota;c__Nitrososphaeria;o__Nitrososphaerales;f__Nitrosopumilaceae;g__Nitrosarchaeum;s__	topology and ANI
Verrucomicrobia	Akkermansiaceae-Baikal1600m-PB-G56		--	4,626,373	33	507,249	33,476	54.4	4,879	271.2	68	86	77.91	3.17	d__Bacteria;p__Verrucomicrobiota;c__Verrucomicrobiae;o__Verrucomicrobiales;f__Akkermansiaceae;g__UBA1315;s__	topology and ANI

*Compleneteness and contamination measured using a custom dataset for Ca. Patescibacteria

Table S3. Genomic parameters of LAGs recovered in this study with ANI > 99.5% to MAGs retrieved from Lake Baikal 1250 and 1350 m deep.

Taxonomy	MAG	Genome Size (bp)	Number of Contigs	Longest Contig Size (bp)	CheckM Completeness (%)	Checkm Contamination (%)
Acidobacteria	Vicinamibacterales-Baikal1600m-PB-G1	4,886,496	43	733,256	92.4	4.8
	Acidobacteria Baikal-deep-G12	3,248,293	182	109,448	79.2	4.3
Actinobacteria	Ca. Planktophila-Baikal1600m-PB-G5	1,448,997	10	388,832	81.2	0.5
	Ca. Planktophila Baikal-deep-G30	1,083,458	58	84,945	72.9	0.5
	Thermoleophilia-Baikal1600m-PB-G15	1,630,020	3	840,662	89.9	0.9
	Thermoleophilia Baikal-deep-G28	1,496,510	19	226,797	85.8	0.0
	Thermoleophilia-Baikal1600m-PB-G16	2,256,336	1	2,256,336	98.4	0.4
	Thermoleophilia Baikal-deep-G21	1,496,510	19	226,797	83.3	0.7
	Ilumatobacteraceae-Baikal1600m-PB-G11	2,345,733	11	855,656	94.7	0.5
	Ca. Nanopelagicales Baikal-deep-G14	1,686,946	104	75,118	86.1	0.8
	Acidimicrobiia-Baikal1600m-PB-G2	2,144,435	8	1,670,992	77.8	1.3
	Acidimicrobia Baikal-deep-G23	2,252,929	180	52,426	85.3	1.3
	Ilumatobacteraceae-Baikal1600m-PB-G10	2,120,470	3	1,629,087	97.9	1.3
	Ilumatobacteraceae Baikal-deep-G17	1,762,442	73	82,555	85.9	6.2
	Ca. Nanopelagicus-Baikal1600m-PB-G4	1,215,619	6	725,322	79.8	0.0
	Ca. Nanopelagicales Baikal-deep-G27	1,081,859	71	49,511	70.6	0.1
	Acidimicrobiia-Baikal1600m-PB-G3	2,083,303	11	823,156	96.6	2.7
	Acidimicrobiia Baikal-deep-G22	1,765,868	93	64,582	96.6	2.1
Alphaproteobacteria	Ca. Fonsibacter-Baikal1600m-PB-G20	946,322	4	627,042	75.5	0.0
	Ca. Fonsibacter Baikal-deep-G36	997,510	66	66,508	95.2	3.7
	Pseudolabrys-Baikal1600m-PB-G21	2,738,833	15	461,811	86.3	0.8
	Pseudolabrys Baikal-deep-G38	2,494,857	119	94,749	88.2	1.3
	Acetobacteraceae-Baikal1600m-PB-G19	3,427,791	9	1,497,302	98.5	2.0
	Acetobacteraceae Baikal-deep-G58	1,946,587	55	177,716	63.1	0.0
Bacteroidetes	Bacteroidetes-Baikal1600m-PB-G24	1,675,431	10	367,283	87.7	0.0
	Bacteroidetes Baikal-deep-G73	1,192,243	86	50,581	76.2	1.4
Gammaproteobacteria	Methyloglobulus-Baikal1600m-PB-G30	2,053,671	12	506,236	95.3	1.3
	Methyloglobulus Baikal-deep-G142	1,106,434	124	32,022	52.6	0.0
	Methylopumilus-Baikal1600m-PB-G31	1,302,697	3	641,110	95.6	0.0
	Methylopumilus planktonicus Baikal-deep-G83	1,171,301	35	110,517	94.2	0.0
	Burkholderiales-Baikal1600m-PB-G29	2,746,740	11	863,158	96.8	2.8
	Betaproteobacteria Baikal-deep-G89	2,062,253	128	76,030	82.5	3.2
Patescibacteria	Ca. Levybacteria-Baikal1600m-PB-G34	907,563	1	907,563	97.7*	0.0*
	Levybacteria-Baikal-deep-G183	677,466	13	174,588	97.7*	0.0*
	Ca. Paceibacteria-Baikal1600m-PB-G35	1,059,132	6	755,885	93.0*	0.0*
	Parcubacteria-Baikal-deep-G184	793,660	24	89,033	90.7*	0.0*
	Ca. Gracilibacteria-Baikal1600m-PB-G33	919,098	17	231,563	67.4*	4.98*
	Paceibacteria-bacterium-Baikal-deep-G197	321,018	41	14,845	39.5*	0.0*
	Ca. Paceibacteria-Baikal1600m-PB-G36	582,052	1	582,052	97.7*	0.0*
	Paceibacteria-bacterium-Baikal-deep-G200	369,798	36	28,033	69.7*	0.0*
	Ca. Saccharibacteria-Baikal1600m-PB-G46	724,546	1	724,546	100.0*	0.0*
	Patescibacteria-bacterium-Baikal-deep-G192	511,372	39	42,074	93.0*	0.0*
	Patescibacteria-Baikal1600m-PB-G49	737,753	1	737,753	97.7*	0.0*
	Patescibacteria-bacterium-Baikal-deep-G193	578,258	29	53,386	72.1*	0.0*
	Ca. Paceibacteria-Baikal1600m-PB-G38	803,329	1	803,329	97.7*	0.0*
	Kaiserbacteraceae-bacterium-Baikal-deep-G195	620,591	49	46,288	62.8*	4.65*
	Ca. Staskawiczbacteria-Baikal1600m-PB-G48	522,042	2	500,723	90.7*	0.0*
	Staskawiczbacteriaceae-bacterium-Baikal-deep-G187	721,116	72	32,480	72.1*	11.6*
	Ca. Paceibacteria-Baikal1600m-PB-G40	592,655	5	171,467	74.4*	0.0*
	Parcubacteria-Baikal-deep-G185	527,091	29	63,755	79.1*	0.0*
	Ca. Paceibacteria-Baikal1600m-PB-G42	558,371	2	485,322	86.1*	0.0*
	Paceibacteria-bacterium-Baikal-deep-G202	465,688	24	52,092	100*	0.0*
	Ca. Paceibacteria-Baikal1600m-PB-G43	754,737	1	754,737	97.7*	2.3*
	Taylorbacteraceae-bacterium-Baikal-deep-G189	685,593	28	65,363	65.1*	2.3*
Planctomycetes	Zavarzinella-Baikal1600m-PB-G53	5,219,492	29	457,339	75.3	4.6
	Gemmataceae Baikal-deep-G171	5,712,728	169	175,789	93.0	4.7
	Gemmataceae-Baikal1600m-PB-G50	4,600,205	10	1,108,979	97.2	1.1
	Gemmataceae Baikal-deep-G161	4,189,651	99	410,398	97.0	1.1
	Gemmataceae-Baikal1600m-PB-G51	4,632,407	21	804,744	93.2	1.1
	Gemmataceae Baikal-deep-G173	2,760,053	276	37,240	86.4	2.3
Thaumarchaeota	Nitrosarchaeum-Baikal1600m-PB-G54	1,280,083	4	793,988	99.0	2.4
	Nitrosarchaeum Baikal-deep-G181	957,170	79	44,673	71.7	1.0
	Nitrosarchaeum-Baikal1600m-PB-G55	1,245,765	2	764,480	99.0	1.0
	Nitrosarchaeum Baikal-deep-G180	716,049	83	55,419	68.0	0.0

PacBio assembled genomes with a better recovery (as indicated by the CheckM completeness) are colored in green, otherwise they are red colored.

*Compleneteness and contamination measured using a custom dataset for Ca. Patescibacteria

Table S4. Genomic parameters of the resulting bins from the Baikal 1600 m CCS sequences. The four Baikalibacteria bins are highlighted in yellow.

Read Binning	Total Size (Mb)	#Reads grouped	Total Deep Baikal 16S rRNA	16S rRNA reads matching CPR (%, total CPR)	Dereplicated Deep Baikal 16S rRNA (97%)	Size after scaffolding (Mb)	Largest Sequence Size and average (Kb)	GC content (± SD)
RBin01	8.77	606	4	1.23	3	--	--	38.7 (±2.4)
RBin02	12.27	838	5	1.54	4	--	--	51.4 (±2.2)
RBin03	7.98	554	6	1.85	2	--	--	45.1 (±5.2)
**RBin04**	**22.35**	**1528**	**36**	**11.11**	**16**	**19.62**	**173 / 16.4**	48.3 (±2.5)
RBin05	11.75	814	6	1.85	1	--	--	35.4 (±1.5)
RBin06	20.98	1575	0	0.00	0	--	--	64.1 (±3.1)
RBin07	7.71	528	8	2.47	3	--	--	39.9 (±1.4)
**RBin08**	**11.15**	**766**	**19**	**5.86**	**9**	**10.73**	**53 / 14.7**	38.6 (±1.4)
**RBin09**	**5.38**	**362**	**22**	**6.79**	**4**	**4.72**	**360 / 16.5**	43.9 (±2.1)
**RBin10**	**24.49**	**1658**	**17**	**5.25**	**10**	**21.31**	**88 / 16.1**	44.0 (±2.0)
RBin11	3.22	218	0	0.00	0	--	--	40.1 (±1.4)
RBin12	3.80	258	2	0.62	2	--	--	56.8 (±1.9)
RBin13	1.43	98	4	1.23	1	--	--	51.3 (±1.6)
RBin14	0.53	38	5	1.54	1	--	--	31.4 (±1.0)
